# Computational design of sequence-specific DNA-binding proteins

**DOI:** 10.1038/s41594-025-01669-4

**Published:** 2025-09-12

**Authors:** Cameron J. Glasscock, Robert J. Pecoraro, Ryan McHugh, Lindsey A. Doyle, Wei Chen, Olivier Boivin, Beau Lonnquist, Emily Na, Yuliya Politanska, Hugh K. Haddox, David Cox, Christoffer Norn, Brian Coventry, Inna Goreshnik, Dionne Vafeados, Gyu Rie Lee, Raluca Gordân, Barry L. Stoddard, Frank DiMaio, David Baker

**Affiliations:** 1 Department of Biochemistry, University of Washington, Seattle, WA USA; 2 Institute for Protein Design, University of Washington, Seattle, WA USA; 3 Department of BioSciences, Rice University, Houston, TX USA; 4 Department of Physics, University of Washington, Seattle, WA USA; 5 Division of Basic Sciences, Fred Hutchinson Cancer Center, Seattle, WA USA; 6 Program in Genetics and Genomics, Duke University, Durham, NC USA; 7 Center for Advanced Genomic Technologies, Duke University, Durham, NC USA; 8 Department of Bioengineering, University of Washington, Seattle, WA USA; 9 Department of Biochemistry, Stanford University School of Medicine, Palo Alto, CA USA; 10 Department of Medicine, Division of Hematology, Stanford University, Stanford, CA USA; 11 BioInnovation Institute, Copenhagen, Denmark; 12 Howard Hughes Medical Institute, University of Washington, Seattle, WA USA; 13 Department of Computer Science, Duke University, Durham, NC USA; 14 Department of Genomics and Computational Biology, University of Massachusetts Chan Medical School, Worcester, MA USA; 15 Department of Biostatics and Bioinformatics, Duke University, Durham, NC USA; 16 Department of Molecular Genetics and Microbiology, Duke University, Durham, NC USA

**Keywords:** DNA-binding proteins, Computational biology and bioinformatics

## Abstract

Sequence-specific DNA-binding proteins (DBPs) have critical roles in biology and biotechnology and there has been considerable interest in the engineering of DBPs with new or altered specificities for genome editing and other applications. While there has been some success in reprogramming naturally occurring DBPs using selection methods, the computational design of new DBPs that recognize arbitrary target sites remains an outstanding challenge. We describe a computational method for the design of small DBPs that recognize short specific target sequences through interactions with bases in the major groove and use this method to generate binders for five distinct DNA targets with mid-nanomolar to high-nanomolar affinities. The individual binding modules have specificity closely matching the computational models at as many as six base-pair positions and higher-order specificity can be achieved by rigidly positioning the binders along the DNA double helix using RFdiffusion. The crystal structure of a designed DBP–target site complex is in close agreement with the design model and the designed DBPs function in both *Escherichia coli* and mammalian cells to repress and activate transcription of neighboring genes. Our method provides a route to small and, hence, readily deliverable sequence-specific DBPs for gene regulation and editing.

## Abstract (web summary)

The authors develop a computational method to design small DNA-binding proteins (DBPs) that target specific sequences. Designed DBPs show structural accuracy and function in both bacterial and mammalian cells for transcriptional regulation.

## Main

Nature uses a wide diversity of DNA-binding protein (DBP) domains for targeting specific sequences^1^, which are often structurally coupled to each other and to effector regions, conferring enzymatic, binding and regulatory functions^2,3^. Despite intensive study and substantial progress in the in silico prediction of DNA-binding specificities from complex structures^4^, the DNA-binding affinity and specificity of natural proteins remain difficult to predict^5^ and the high free-energetic cost of desolvating the highly polar DNA surface presents a challenge to the de novo design of DBPs. For these reasons, while computational de novo design has had considerable recent success in generating binders to arbitrary protein structures^6^, mostly at hydrophobic patches, computational approaches for DBP engineering have thus far been limited to redesigning interfaces of existing native protein–DNA complex structures^7–11^. These efforts have been constrained by the rigid geometry of the starting scaffold shape and orientation relative to DNA^12^, which restrict the possible target sequences that can be recognized^13^. A more general solution to generating compact, customizable DBPs would enable modular, geometrically precise and deliverable tools and be highly complementary to state-of-the-art techniques in gene regulation, gene editing and nucleic acid diagnostics, which primarily use Cys_2_His_2_ zinc finger (ZF) domains^14,15^, transcription activator-like effectors (TALEs)^16,17^ and CRISPR–Cas^18^. While these tools have proven powerful, each has limitations. ZFs can be laborious to engineer and the size of TALE and CRISPR–Cas systems complicates their delivery in therapeutic applications; CRISPR–Cas systems also require an extra guide RNA component and target sites are constrained by protospacer-adjacent motif requirements^18^. These systems will undoubtedly continue to be improved but their constrained backbone topologies can limit precise control of interaction specificity and close integration with diverse effector domains.

## Results

### Design strategy

**Fig. 1:**
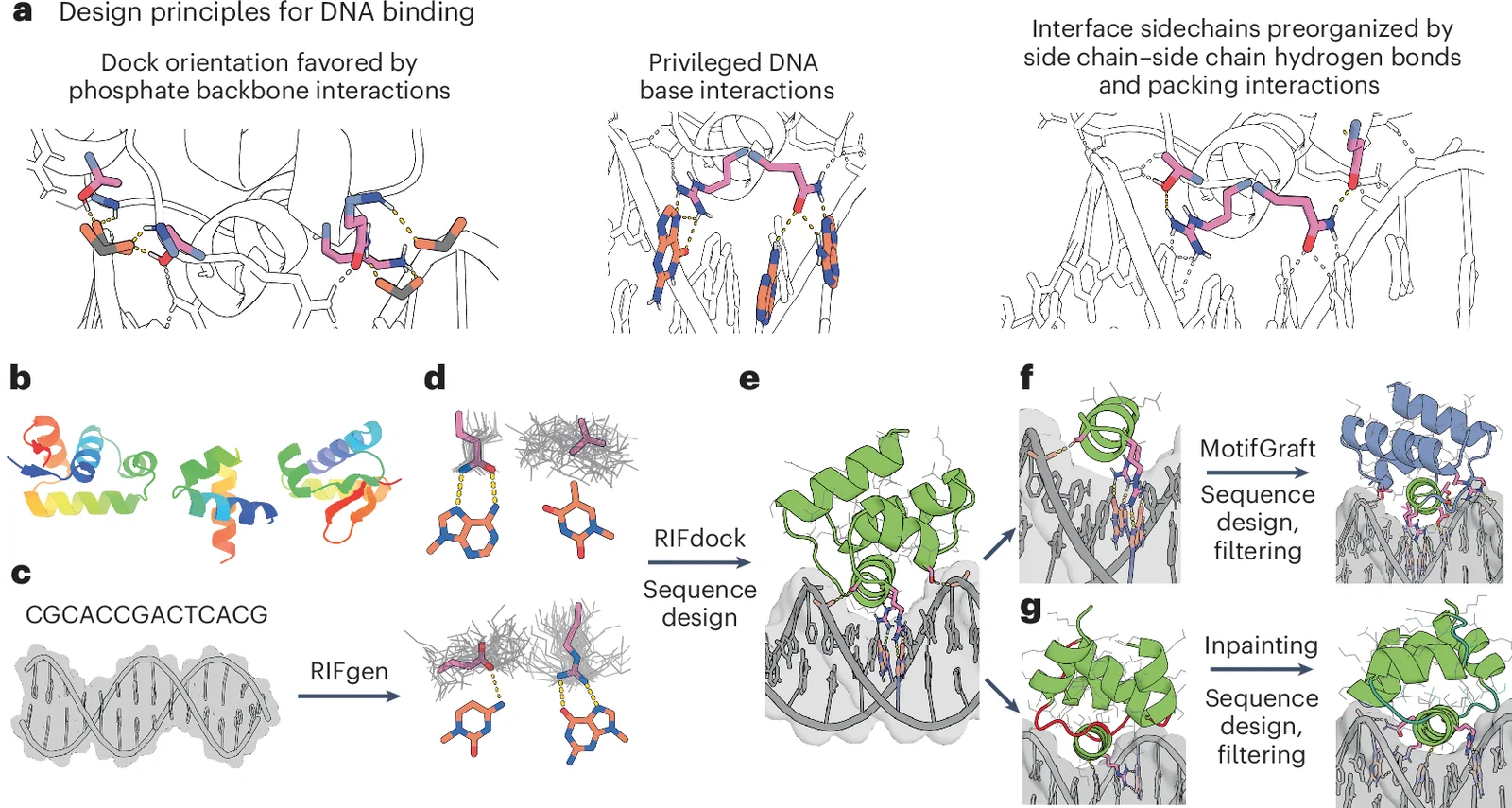
Overview of the DNA binder design pipeline. **a**, Design principles for design of sequence-specific DBPs. **b**, HTH backbone scaffold library generated from metagenomic sequences. **c**, DNA target, starting with either a specific nucleotide sequence modeled as B-DNA or a DNA crystal structure. **d**, Generation of RIF (gray) to form base-specific hydrogen bonds and hydrophobic packing interactions. Example rotamers (pink) are generated for nucleotide bases (orange; clockwise from top left: adenine, thymine, guanine and cytosine). **e**, Docking of scaffolds onto the RIF to identify seed interactions and placements with base-specific contacts, followed by sequence optimization of the DNA–scaffold interactions using Rosetta or LigandMPNN-based sequence design and Rosetta modeling. **f**, Recognition helices making multiple favorable interactions with the target are extracted from first-round designs and grafted onto the scaffold library, followed by further rounds of interface sequence design and filtering for favorable interactions. **g**, Inpainting of the protein loops (red) results in new connecting loops (teal) between the helical portions of the design, followed by further rounds of interface sequence design and filtering.

We reasoned that it would be possible to achieve DNA sequence recognition using small, compact proteins by sampling a wide variety of structures and binding modes to find those that are optimal for targeting specific sequences of interest. We previously developed a general method for designing specific protein binders to arbitrary protein targets on the basis of this concept^6^ but sequence-specific DNA binding requires overcoming several additional challenges. First, binding the DNA double helix, with major and minor grooves, requires sufficient shape complementarity with the DNA backbone to precisely position specific amino acid residues to interact with the DNA base edges. Second, recognition of DNA sequences requires distinguishing between the subtle changes in individual atom placements among the four bases^19–25^, which alter the landscape of potential molecular contacts. Third, in contrast to designed protein–protein contacts mostly mediated by orientation-agnostic hydrophobic patches^6^, the majority of accessible DNA base atoms require hydrogen-bond interactions with polar side chains for specific recognition^26^. Not only are polar interactions harder to model accurately but the longer polar side chains have considerable conformational flexibility, making structure modeling more difficult and increasing opportunities for off-target base interactions through alternate side-chain rotamer conformations. To address these challenges, we formulated a set of design principles (Fig. 1a) and sought to develop a design pipeline implementing them in the context of small helical DBP domains that target short DNA sequences (Fig. 1b–g).

For design challenge 1—positioning protein scaffolds so that amino acid sidechains can contact the DNA bases—we hypothesized that interactions with the phosphate backbone, such as the backbone amide-mediated hydrogen-bond interactions with DNA phosphate oxygens (hereon called main-chain phosphate hydrogen bonds) that are frequently observed in native DBP structures, could enable precise placement of designed scaffolds such that residues designed to make specific base contacts interact in the intended geometry. We reasoned that such satisfaction of the hydrogen-bond requirements of the DNA backbone phosphates and the DNA bases would substantially constrain viable design geometries. We hypothesized that the helix–turn–helix (HTH) DNA-binding domain would be a good candidate for computational DNA binder design as it is relatively small and compact and is capable of making direct contacts with DNA through a recognition helix within the DNA major groove^27^. To generate a library of small (<65 aa) and structurally diverse scaffolds, we took advantage of the vast amount of metagenome sequence data and the accuracy of deep-learning-based protein structure prediction (Supplementary Fig. 1). We carried out sequence searches for HTH DNA-binding domains, generated AlphaFold2 (AF2) structure predictions^28^ and filtered these on the basis of prediction confidence (predicted local distance difference test, pLDDT) and template modeling score (TMscore) to known HTH domain structures^29^. This resulted in a library of ~26,000 HTH scaffolds, which finely sample different helix orientations and loop geometries (Supplementary Fig. 1 and Methods).

We docked the scaffolds against specific DNA target structures seeking to maximize the potential for specific side chain–base interactions (Fig. 1b–d). To do this, we extended the RIFdock approach^6^ to protein–DNA interactions (Methods), which finely samples many possible de novo docks for each scaffold. RIFdock begins by enumerating a large and comprehensive set of disembodied side-chain interactions, called a rotamer interaction field (RIF), that make favorable interactions with the desired target. We focused RIF generation on polar and nonpolar interactions with nucleotide base atoms in the major groove of the DNA target, with an emphasis on protein side chain–DNA base hydrogen-bonding interactions that are statistically more probable in native protein–DNA complexes^30^. In RIFdock, we constrained the RIF DNA base-specific interactions to the HTH recognition helix to find placements with both main-chain phosphate hydrogen bonds and base-contacting RIF sidechains, resolving the first design challenge.

To address design challenge 2—recognizing specific DNA bases—we used either Rosetta-based sequence design or an extended version of the deep-learning-based ProteinMPNN sequence design software (Fig. 1e and Methods). The ProteinMPNN graphical model generates amino acid sequences purely on the basis of protein backbone coordinates and a recent extension to incorporate ligand and DNA atoms in the interaction graph, called LigandMPNN^31^. While the Rosetta-based sequence design protocol was constrained by a position-specific scoring matrix (PSSM) for each scaffold, LigandMPNN was purely based on the structure of the designed complex. To reduce the computational cost of full sequence design on the millions of generated scaffold docks for each target site, we first repacked only the RIF side-chain residues in the context of the target to remove potential clashes between designed side chains. Docks for which good protein–DNA interactions could be achieved without side-chain clashes were then subjected to multiple iterations of full sequence design, alternating with Rosetta backbone relaxation to maximize complementarity to the target sequence. We generated 200,000–300,000 designed complexes per target. From this large set of designs, we selected those with the most favorable free energy of binding (Rosetta *ΔΔG*), contact molecular surface area^6^ and interface hydrogen bonds, with the fewest interface buried unsatisfied hydrogen-bond donors and acceptors and with bidentate side chain–base hydrogen-bonding arrangements frequent in the Protein Data Bank (PDB) (Methods).

To address design challenge 3—precise geometric side-chain placement—we hypothesized that specificity and affinity would be improved in designs with highly preorganized interface side chains. We reasoned that preorganization would be especially important for long polar side chains with many possible conformations. We achieved preorganization through side chain–side chain hydrogen bonding and assessed it using the Rosetta RotamerBoltzmann calculation^32^. By selecting only designs with native-like preorganization of key contacts (Supplementary Fig. 2), we aimed to achieve the level of precision required for specific DNA binding.

Following selection based on the above criteria and clustering by sequence identity, the monomeric structures of the hundreds to thousands of remaining designs for each target were predicted on the basis of their sequences using AF2 and designs that deviated from their original design models were discarded. The remaining predicted monomer structures were superimposed onto the design complex through alignment on the interface residues of the original design and relaxed with Rosetta in the context of the DNA. Designs with the most favorable DNA-binding interactions after superimposition, as assessed with the above metrics, were selected for experimental characterization. To obtain additional high-quality designs, the DNA-interacting segments of the filtered designs were extracted, clustered and grafted back into the original in silico scaffold library, followed by a second round of sequence design (Fig. 1f)^6^. We also diversified the best designs using RoseTTAFold Inpainting^33^, focusing on the resampling of scaffold loops, followed by sequence design (Fig. 1g). We generated at least 10,000 designs for each DNA target that passed all the structural and DNA interaction filters using a combination of these approaches.

### DBP generation and screening with yeast display cell sorting

We created three sets of designs using variations of the overall design approach. In the first set, we generated 21,488 designs using Rosetta-based sequence design, the motif grafting strategy and our custom scaffold library of AF2-predicted native DNA-binding domains. In this set, the double-stranded DNA (dsDNA) targets were the DNA portions of cocrystal structures. In the second design set, we generated 12,273 designs against the same DNA sequences with the LigandMPNN sequence design strategy and the motif grafting approach for backbone resampling. In this case, rather than designing only against the dsDNA conformations found in each target’s respective crystal structure, we also designed against straight B-DNA of the same sequences (6,608 for B-DNA and 5,666 for crystal-derived DNA). The LigandMPNN approach was less effective at generating designs with a high contact molecular surface, likely because of the ability of Rosetta to relax the protein backbone during sequence design, but ultimately produced designs with more favorable free energy of binding (Rosetta *ΔΔG*) and an increased number of hydrogen bonds to bases (Supplementary Fig. 3). Lastly, in the third set we generated 100,000 designs using the LigandMPNN-based design pipeline and the inpainting-based backbone remodeling protocol against 11 unique B-DNA targets. To test whether our method could generate binders to novel DNA sequences, design set 3 sequences were not derived from a crystal structure and contained submotifs not represented among DNA sequences bound by protein–DNA complexes in the PDB or in the JASPAR nonredundant transcription binding profile database^34,35^.

For each set of designs, synthetic oligonucleotides (230 bp) encoding the 50–65-aa designed proteins were ordered in a single pool and cloned into a yeast surface-expression vector. Cells containing designs that bound each DNA target were enriched by several rounds of fluorescence-activated cell sorting using fluorescently labeled target dsDNA oligos. The naive and sorted populations for each DNA target were deep-sequenced and the frequency of each design in the starting population and after each sort was determined. From this analysis, we identified 97 designs that were substantially enriched (>100×) in pools sorted with their intended dsDNA target compared to the naive library.

**Extended Data Fig. 1:**
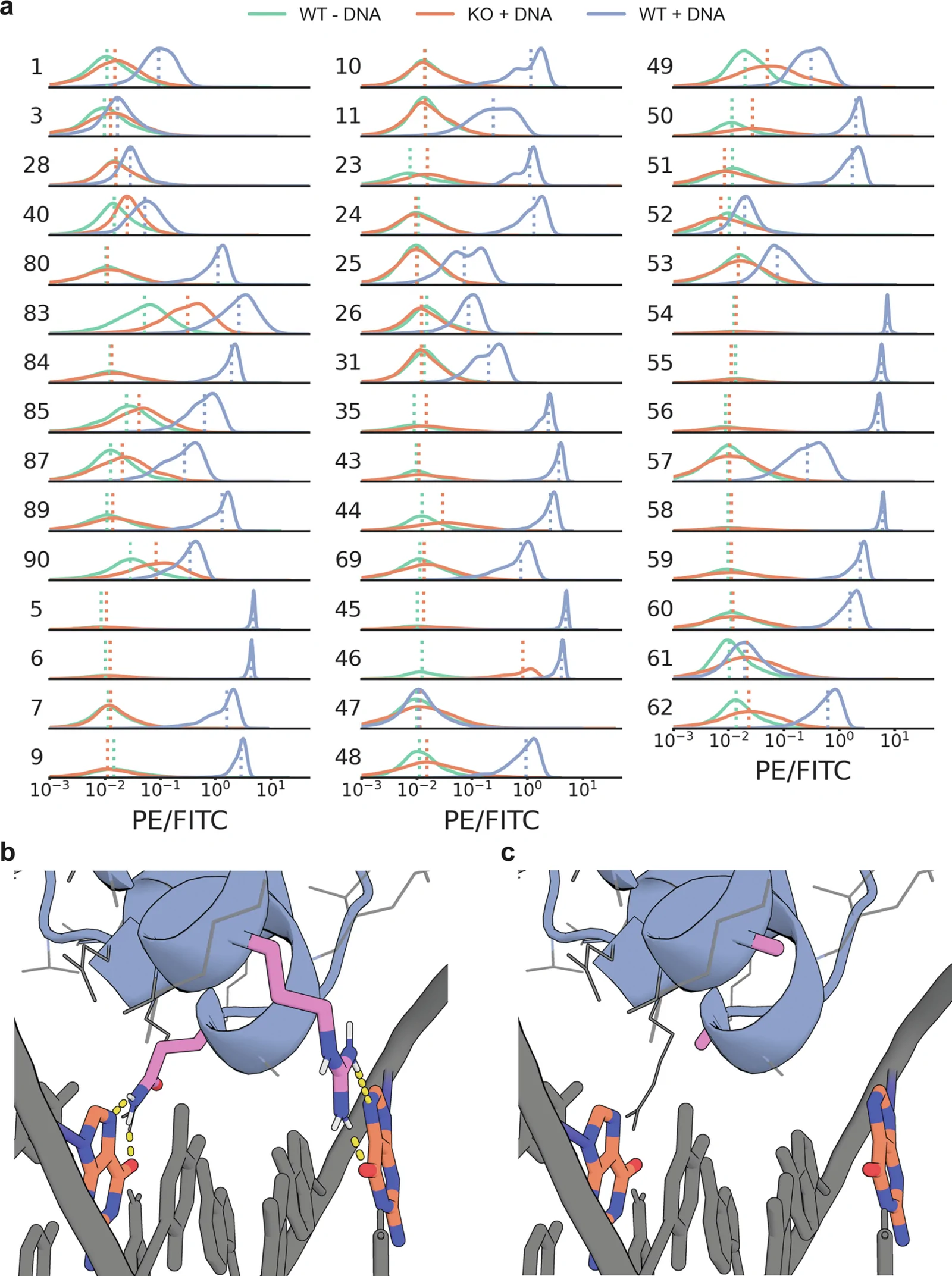
Clonal analysis of binder designs by yeast surface display confirms dsDNA-binding function. **a**, Histograms of binding activity (PE/FITC) are shown for each design. Knockout sequences were created by mutating 1–3 key interface residues for base-specific contacts present in the wildtype (WT) design model (Supplementary Table 1). Samples of the WT design (WT + DNA, blue), and the knockout sequence (KO + DNA, orange) with target were analyzed after labeling with each respective dsDNA oligo at 1 µM with avidity (DBPs 7, 10, 11, 24, 25, 26, 28, 31, and 40 collected without avidity). The background signal of the wildtype design without dsDNA labeling (WT-DNA) is shown in green. Interface knockouts substantially disrupted dsDNA-binding in nearly all cases. **b**, Example (DBP43) of interface knockout of the original design model with base-specific hydrogen bonding ARG and GLN residues (pink). **C**, Model showing the two ALA substitutions (pink) of those residues. Source data

We tested these 97 designs as individual clones in a 96-well screening format and found detectable binding for 44 of them (Extended Data Fig. 1). The remainder may result from doublet transformants in the yeast pool or are very weak binders that were enriched under higher dsDNA oligo concentrations. Of the 44 successful designs, 30 were derived with targets modeled as ideal B-DNA and 14 were derived with DNA crystal structure models as targets. For each of these designs, we knocked out the DNA-binding interface by substituting the 2–3 residues making the most extensive interactions with the DNA bases (Supplementary Table 1). These knockout mutations completely or substantially disrupted binding for all designs that had detectable binding on yeast (Extended Data Fig. 1), indicating that the functional designs worked as intended.

### Design conformation and binding specificity

**Fig. 2:**
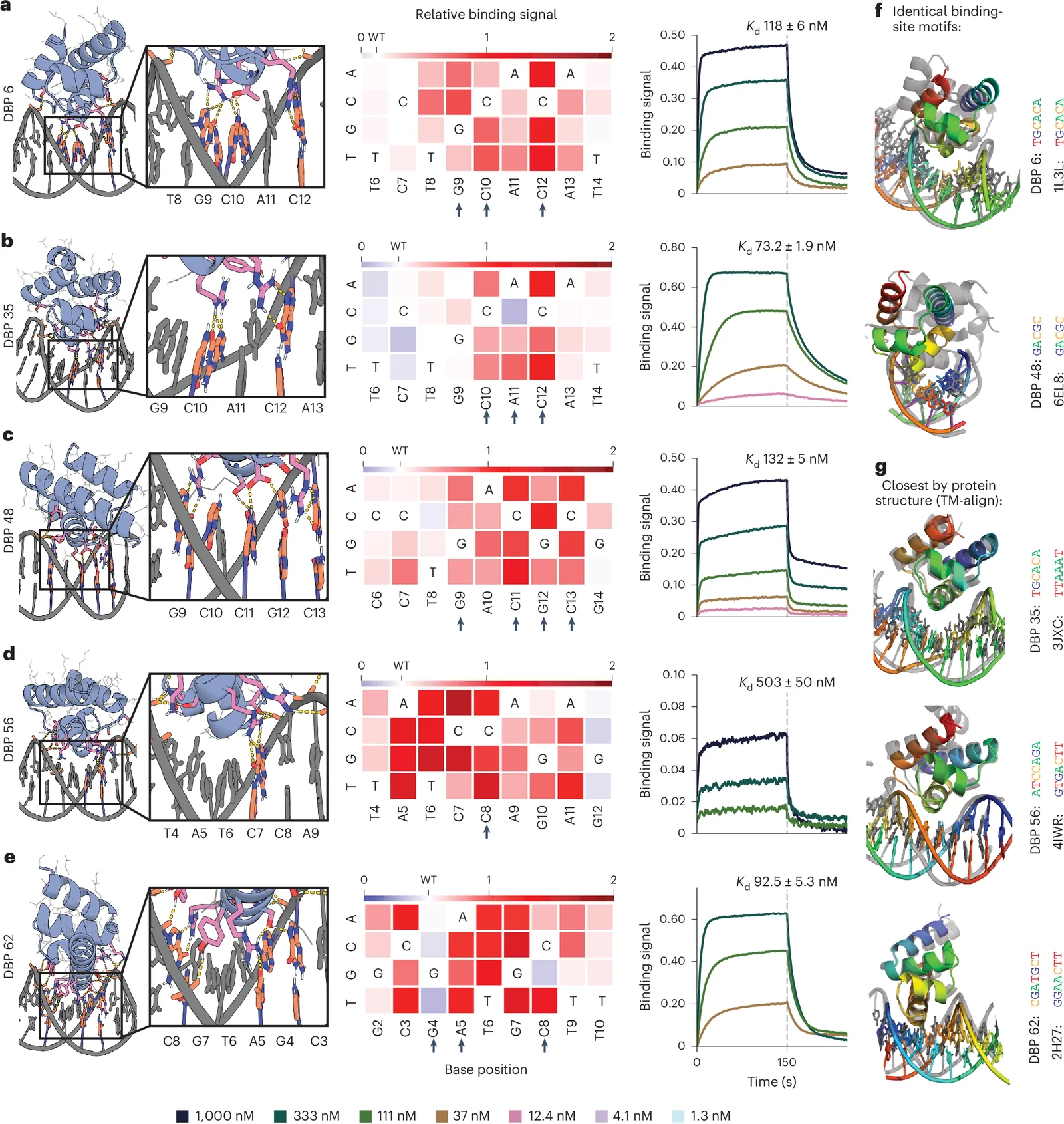
Designed DBPs bind with high affinity and specificity to their intended target sites. **a**–**e**, Characterization of DBPs 6 (**a**), 35 (**b**), 48 (**c**), 56 (**d**) and 62 (**e**). Left: computational design models of characterized designs at the DNA-binding interface. DNA bases and protein residues involved in hydrogen-bonding interactions are shown in orange and pink, respectively. Hydrogen bonds are highlighted with dashed yellow lines. Middle: relative binding activity (PE/FITC normalized to the no-competitor condition) from flow cytometry analysis in yeast display competition assays with all possible DNA base mutations at each position of the competitor oligo. Blue indicates competitor mutations where competition was stronger than with the wild-type (WT) competitor, while red indicates competitor mutations where competition was weaker. Arrows indicate base-pair positions contacted with hydrogen bonds or hydrophobic contacts to base atoms in the design model. Additional characterized designs are shown in Extended Data Fig. 3. Right: binding of purified miniprotein designs to the DNA target with BLI. Each line represents biotinylated dsDNA target dilutions by 1:3. The highest DNA target concentration is indicated in each plot. Additional characterized designs are shown in Extended Data Fig. 5. **f**, DBPs 6 and 48 (colored) differ in both structure and docking mode to native cocomplex structures with matching DNA-binding sites (gray). **g**, DBP 35 has a similar structure and dock to the closest match in the PDB but binds a distinct DNA target site, whereas DBPs 56 and 62 have structures similar to the closest matches but different docks and DNA target sites. DBP 48 was analyzed with sequence C because of its improved binding signal and nearly identical modeled binding sites (Extended Data Fig. 2); all other designs were analyzed with their designed target sequence. Source data

**Extended Data Fig. 2:**
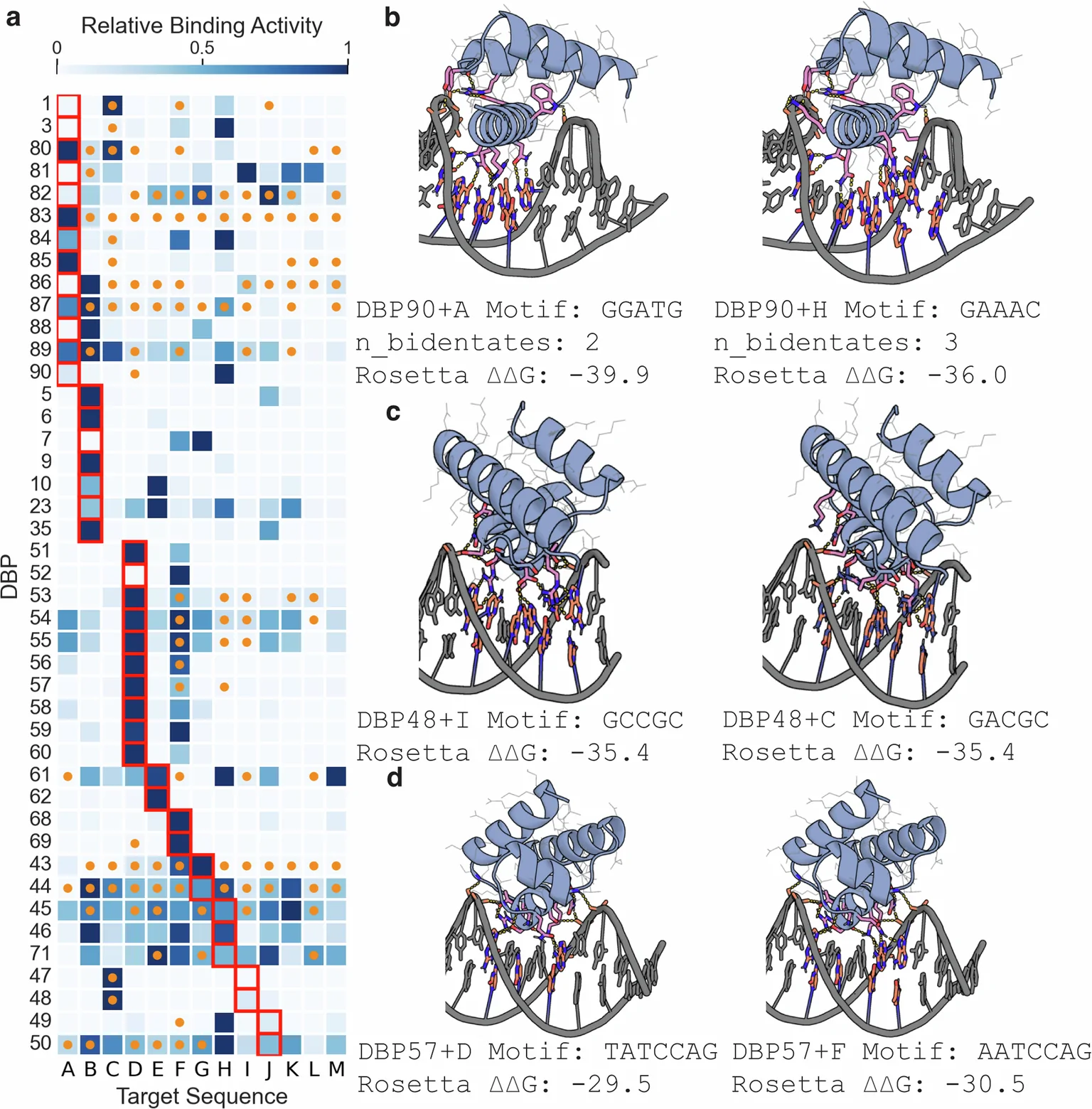
All-by-all analysis of selected designs by yeast surface display reveals preferential target binding of designs. **a**, Yeast surface display relative binding activity (Normalized PE/FITC) of each design labeled at 1 µM dsDNA with avidity, normalized by design row. Red squares indicate the intended target sequence for each design. Orange dots indicate target sequences containing Rosetta-predicted binding motifs. Sequences were considered potential binding targets if they had Rosetta ΔΔG less than or equal to the designed complex. DBPs 83, 85, 65, 6, 9, 35, 69, 47, 48, 51, 56, 57, 60, and 62 were considered to preferentially bind less than 3 of the 13 tested DNA target sequences, including their designed target sequence. **b**, DBP90 bound weakly to its initial design target, but strongly to an alternate target sequence (H) with slightly higher Rosetta ΔΔG but also allowed for bidentate hydrogen bonds to 3 bases. Left: DBP90 + A (on-target model), Right DBP90 + H (alternate target model). **c**, DBP48 bound weakly to its initially designed target sequence, but strongly to Rosetta-predicted alternative target site (D) that differed by only 1 base pair across the interface and had equivalent Rosetta ΔΔG. Left: DBP48 + I (on-target model), Right DBP48 + D (alternate target model). **d**, DBP57 bound strongly to its initial design target as well as an alternate target that contained an identical 6 bp stretch (ATCCAG) at the binding interface. Left: DBP57 + E (on-target model), Right DBP57 + C (alternate target model). Source data

We performed an all-by-all screen of DBP design hits to 13 unique dsDNA targets (Extended Data Fig. 2 and Supplementary Table 2). Several designs exhibited a strong preference for only their designed target sequence (for example, DBPs 6, 9 and 62), others exhibited a strong preference for two or three of the sequence targets (for example, DBPs 1, 52 and 60) and a few bound to most of the targets (for example, DBPs 23, 44 and 89). To try to understand these observed binding preferences, each tested DNA sequence was threaded onto each design complex model at all possible base-pair alignments, the alternative complex models were relaxed with Rosetta and the model with the most favorable Rosetta *ΔΔG* was selected. We found a modest correlation between the predicted free energy of binding and the extent of off-target binding (Extended Data Fig. 2); for DBPs 44 and 89, Rosetta *ΔΔG* values comparable to the original targeted sequence were obtained for most of the off-target sites, consistent with the observed low specificity. Overall, we found that 14 designs bound with specificity closely consistent with the design models (DBPs 5, 6, 9, 35, 43, 69, 47, 48, 51, 56, 57, 60, 62 and 85), including binders for five unique DNA sequences (sequences A–E). Specific binders were obtained for some sequence targets, such as sequence D, at much higher rates than others, suggesting a preference for specific DNA motifs. Indeed, many of the sequence D binder design models contain very similar interface hydrogen-bond contacts (Fig. 2d). This may reflect a greater suitability of HTH scaffolds for some motifs over others, the specific DNA shape formed by the preferred target motifs or an inherent preference of the LigandMPNN model.

**Extended Data Fig. 3:**
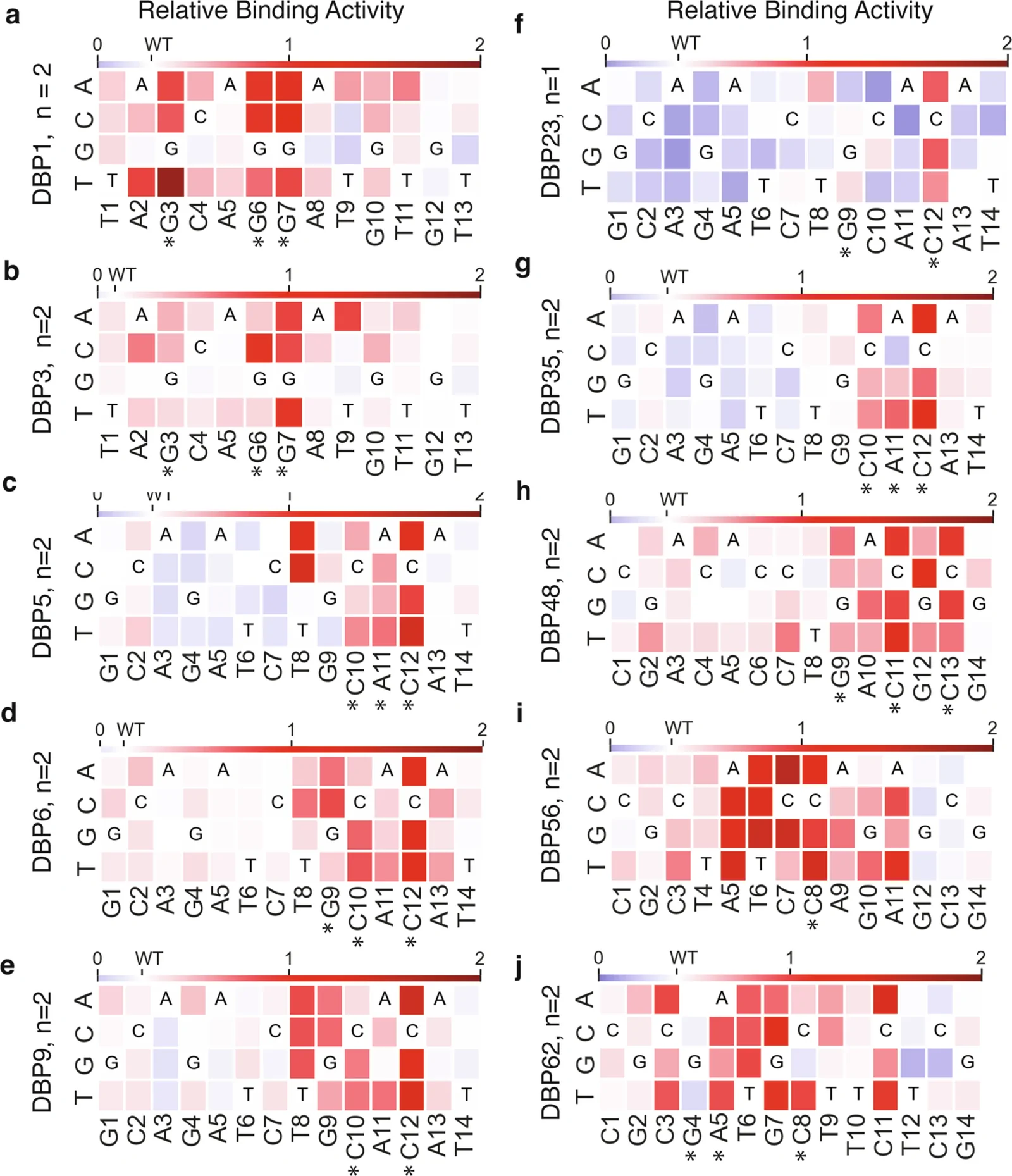
Full competition assays for all DBPs designs. **a-j**, Relative binding activity (Median PE/FITC normalized to the no-competition sample) from flow cytometry analysis in yeast display competition assays for designs DBP1, DBP3, DBP5, DBP6, DBP9, DBP23, DBP35, DBP48, DBP56, and DBP62, respectively, with all possible DNA base mutations at each position of the competitor oligo. Heat maps show the mean of both replicates. Blue indicates competitor mutations where competition was stronger than with the wild-type competitor, while red indicates competitor mutations where competition was weaker. Competitor mutations where competition was weak (red) suggest incompatibility with binding to the competitor oligo. Asterisks indicate base pair positions contacted with hydrogen bonds or hydrophobic contacts to base atoms in the design model. Additional plots for individual replicate experiments are included in the raw data. DBP48 was analyzed with sequence C due to its improved binding signal and nearly identical modeled binding sites. All other designs were analyzed with their designed target sequence. In several cases we observed extra specificity beyond the positions directly involved in hydrogen bonding and hydrophobic contacts. For example, DBPs 6 and 9 exhibit specificity for a 6 nucleotide stretch (TGCACA) with peripheral dependence on T8 and A13. This specificity is most likely explained by effects of shape readout that are not considered by Rosetta modeling of the designs. DBP62 appears dependent on bases peripheral to the binding site (for example C11). Source data

We used a yeast display competition assay to characterize the DNA-binding site specificity of a subset of the designs (Fig. 2a–e, left, and Extended Data Fig. 3). Addition of nonbiotinylated competitor dsDNA to the biotinylated target sequence reduced binding signal by flow cytometry and scanning base substitutions through the competitor revealed positions important for binding (Fig. 2a–e, middle). DBPs 6, 35, 48, 56 and 62 exhibited specificities consistent with the designed side chain–base interactions. For example, in DBP 6, R31 and R36 in the design model form bidentate hydrogen bonds with the guanines of base-pair positions G12 and C9, respectively, while T32 forms a hydrogen bond with C10. Substitution of the bases at positions 9, 10 and 12 eliminated competition, indicating specificity for the GCxG motif as expected (Fig. 2a). DBP 62 exhibited specificity for its target site despite having relatively few base-specific hydrogen-bonding interactions; specificity in this case may have resulted from the very tightly packed interface (Fig. 2e). The observed specificities agree with those predicted from the design model using DeepPBS for DBPs 5, 6, 9 and 35 (ref. ^4^).

**Extended Data Fig. 4:**
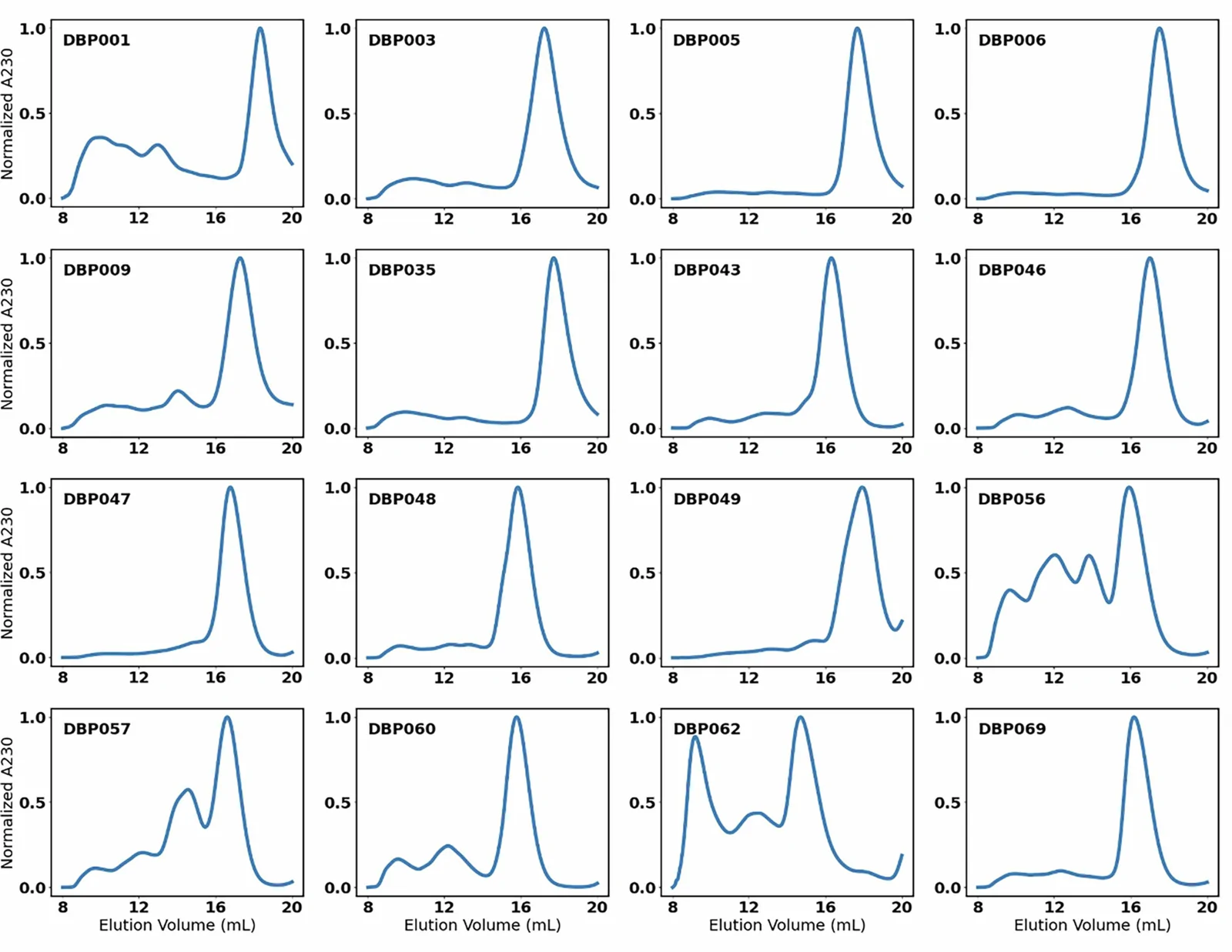
SEC traces of purified proteins. Normalized absorbance at 230 nm of elution over a Superdex™ 75 Increase 10/300 GL column. Each plot shows a separate protein sample, following IMAC purification, with the HIS-tag attached. In every case, the highest peak, corresponding to the protein of interest, was collected and used for *in vitro* experiments. Source data

**Extended Data Fig. 5:**
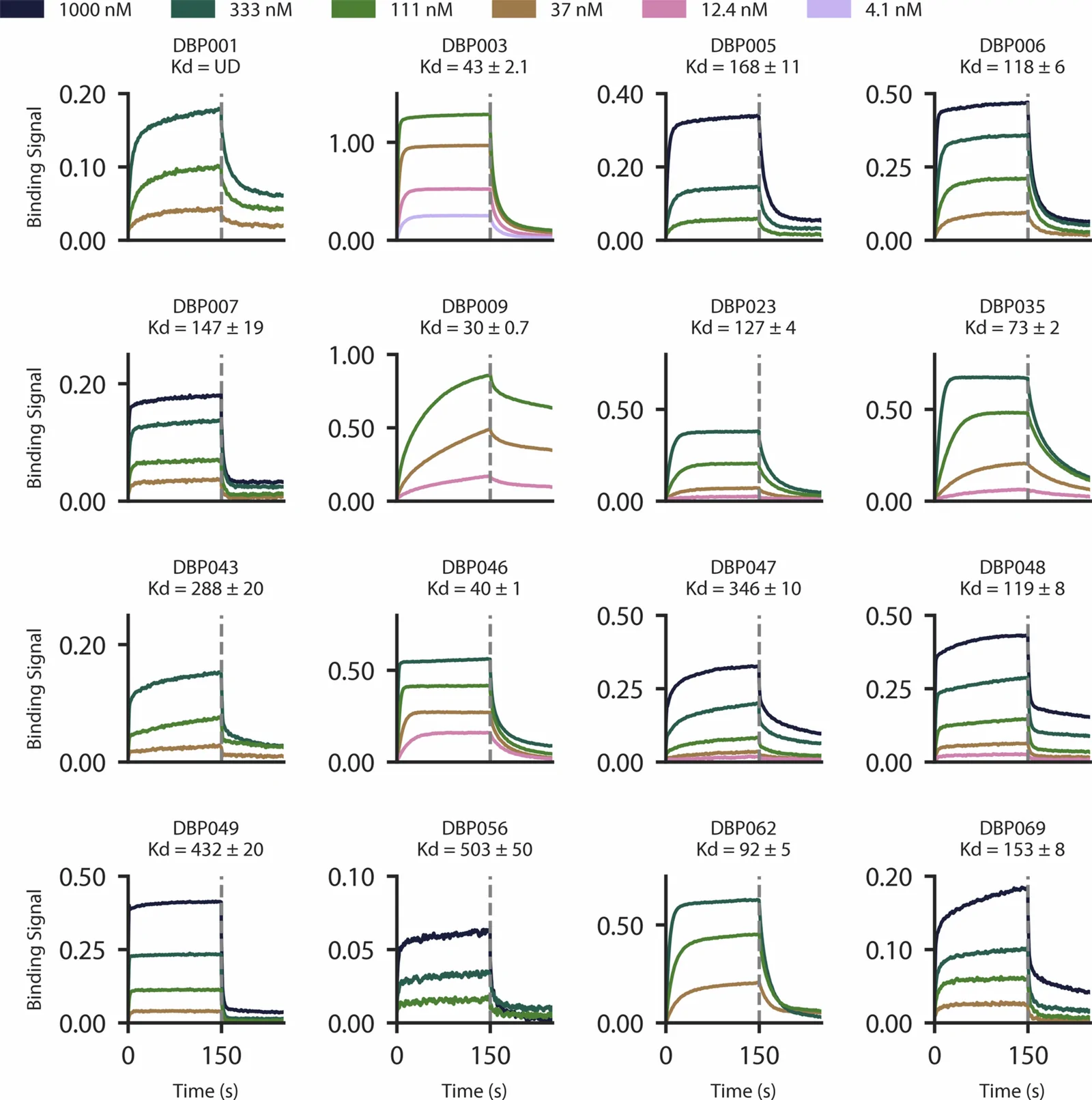
Purified designs bind their respective dsDNA targets *in vitro* by biolayer interferometry. Binding of purified miniprotein designs to the DNA target with BLI. Each line represents biotinylated dsDNA target dilutions by 1/3. K_D_ values are indicated above each plot. UD indicates unable to determine. DBP48 was analyzed with the sequence C dsDNA target. Source data

Genes encoding the designs were encoded for *Escherichia coli* expression and purified proteins were evaluated for binding in vitro. Most of the selected designs were in the soluble fraction, readily purified by Ni^2+^-NTA chromatography, and appeared monodisperse by size-exclusion chromatography (Extended Data Fig. 4). Binding to the biotinylated dsDNA oligo was assessed using biolayer interferometry (BLI) and all designs were found to bind with binding affinities ranging from 30 to 500 nM (Fig. 2a–e, right, and Extended Data Fig. 5).

**Extended Data Fig. 6:**
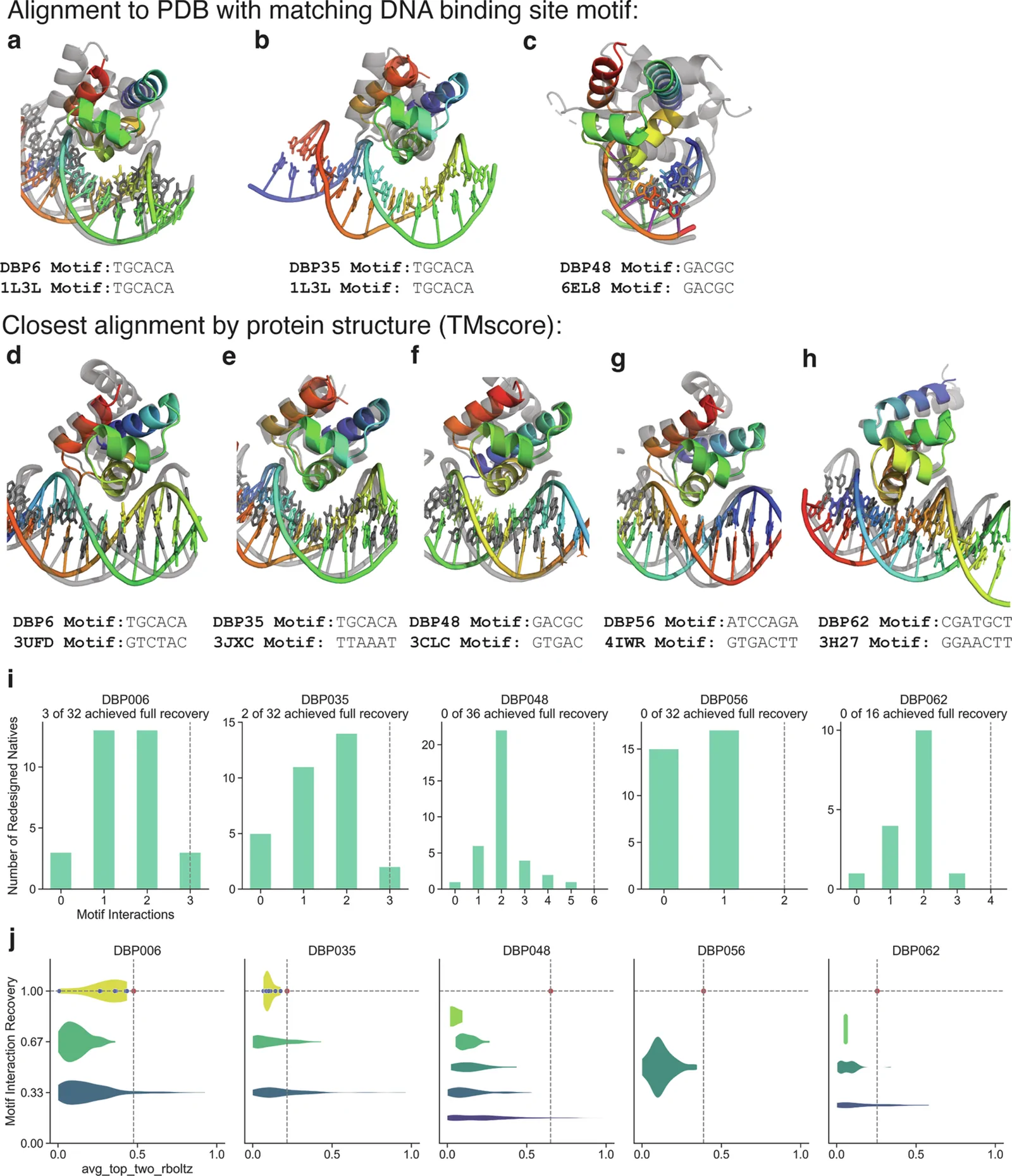
Comparison of designed DBPs with nearest native structures by target motif or protein structure. **a-c**, Alignment of DBP designs to PDB structures containing an identical DNA binding site motif. Native structures are shown in gray aligned to the DBP design (colored). DNA sequence matches were found by creating a set of all contiguous DNA binding site motifs in the PDB where any atom of a protein residue was within 5 Å of an atom in the contiguous DNA sequence motif. **D-h**, Structural alignment of DBP designs to nearest PDB structures by TM-align. TM-align searches were performed on protein-DNA co-complex structures in the PDB to identify the nearest native protein scaffold. Nearest structures are shown in gray aligned to the DBP design (colored). **i**, Computed statistics on native DBPs in the PDB (Supplementary Table 3) redesigned in the presence of the designed DBP’s DNA target motif. We examined whether the same amino acids formed hydrogen bonds with the same DNA base atoms (motif interaction recovery). The native redesign method was able to achieve full motif interaction recovery (dashed line) for DBPs 6 and 35, but not the remainder of analyzed designs. **j**, Analysis of sidechain preorganization for recovered motifs residues by average top two RotamerBoltzmann score. Violin plots show the distribution of *avg_top_two_rboltz* among recovered interacting residues for each design. Individual data points are shown for designs with full motif atom recovery (original design in red, best native redesigns in blue). Source data

Although some of the designs target DNA sequences found in crystal structures, the designed DBPs and their sequence preferences are novel, as assessed by comparison of the binding-site motifs to cocomplex structures of native DBPs in the PDB containing a protein helix in contact with bases in the DNA major groove. We found that some designs (DBPs 6, 35 and 48) preferred a similar motif as native DBP structures but had substantially unique interfaces and docking orientations, while other designs (DBPs 56 and 62) bound novel sequences found neither in the PDB (Fig. 2f and Extended Data Fig. 6a–c) nor in the JASPAR nonredundant transcription binding profile database^34,35^ (Supplementary Fig. 4).

Our binder design method aims to effectively sample diverse scaffold–DNA docks to find solutions optimal for binding the target DNA sequence. The method could, in principle, recover solutions similar to known native DBP–DNA complexes. To investigate this, we compared the structures of our designed DBPs to native DBP domains in DNA cocrystal structures in the PDB by TM-align^29^ (Fig. 2g, Extended Data Fig. 6d–h and Supplementary Table 3). We found that the overall folds of the designed scaffolds had matches in the PDB but the placement of the scaffold relative to the DNA generally differed, as expected given the de novo docking step in our approach. None of the closest matches by protein structure had more than three of seven common bases at the aligned DNA-binding site positions and the side chain–base hydrogen-bond networks differed substantially. For all designs that bound their DNA targets, we also performed BLASTp searches of the nonredundant protein sequences database^36^ and found that most had sequence similarity to native metagenome protein sequences ranging from 40% to 60% (Supplementary Table 1). Overall, these analyses suggest that our approach was able to use and expand upon the known native docking space, while exploring new sequence space, to identify effective DBP designs against the specified target sequences.

To evaluate the importance of backbone sampling through docking, we examined the ability of LigandMPNN-based sequence design to generate interfaces passing our in silico metrics when starting from crystal structures of native cocomplexes rather than de novo docks. Starting from cocrystal structures with high TM-align scores to the designed DBPs, we mutated the DNA sequence in silico to the target sequence and redesigned the sequence using LigandMPNN. We found that designs based on fixed native backbones failed to recover most of the base-specific hydrogen bonds present in the designs produced by our docking pipeline (Extended Data Fig. 6i). In the few cases where native redesign did recover multiple base-specific hydrogen bonds, such as DBPs 6 and 35, the de novo docked design models scored better on side-chain preorganization by the RotamerBoltzmann metric (Extended Data Fig. 6j), suggesting non-hydrogen-bond features of the interface that may be critical for specific binding and require precise docking configurations. Overall, our design method identifies designs that would not be identified through structure-based redesign and generates specific binders for unique DNA sequences that are not known to be recognized by native proteins.

### X-ray cocrystallography and DBP footprinting

**Fig. 3:**
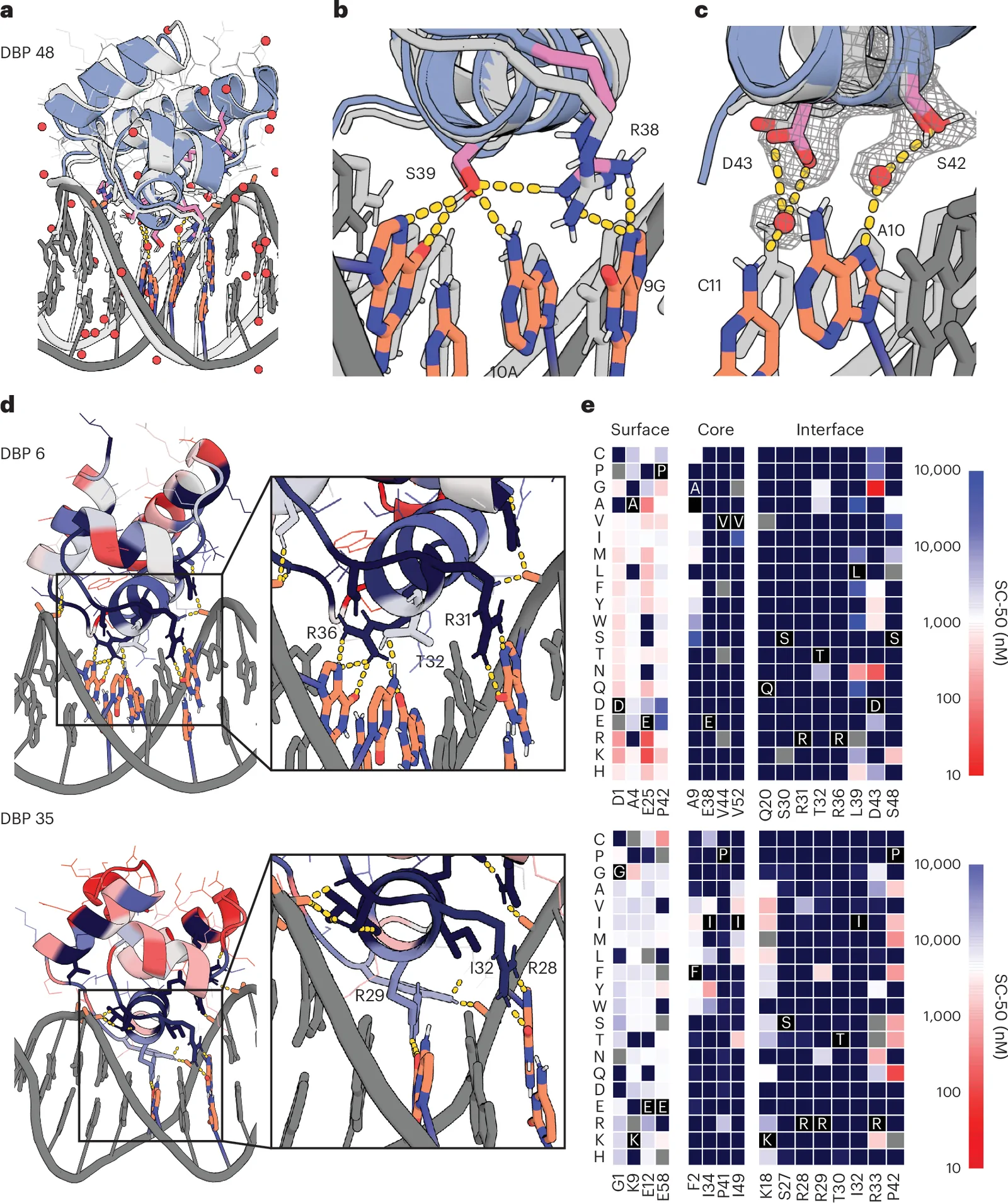
Structural validation of DNA binder designs. **a**, Cocrystal structure of DBP 48 (colored) and the design model (gray) are in close agreement. **b**, Zoomed-in view showing the close agreement of critical interface residues R38 and S39 between the crystal structure and design model. **c**, Close-up view of water-mediated hydrogen bonds formed by S42 and D43. **d**, Left: designed DBPs colored by positional Shannon entropy from SSM, with blue indicating positions of low entropy (conserved) and red indicating positions of high entropy (not conserved). Right: zoomed-in views of central regions of the design interfaces. **e**, Heat maps representing SC_50_ values for single mutations in the design model core (left) and the designed interface (right). Substitutions that are heavily depleted are shown in blue and beneficial substitutions are shown in red. Full SSM maps over all positions and close-up views of DBP 1 are provided in Supplementary Figs. 5 and 6. Source data

**Extended Data Fig. 7:**
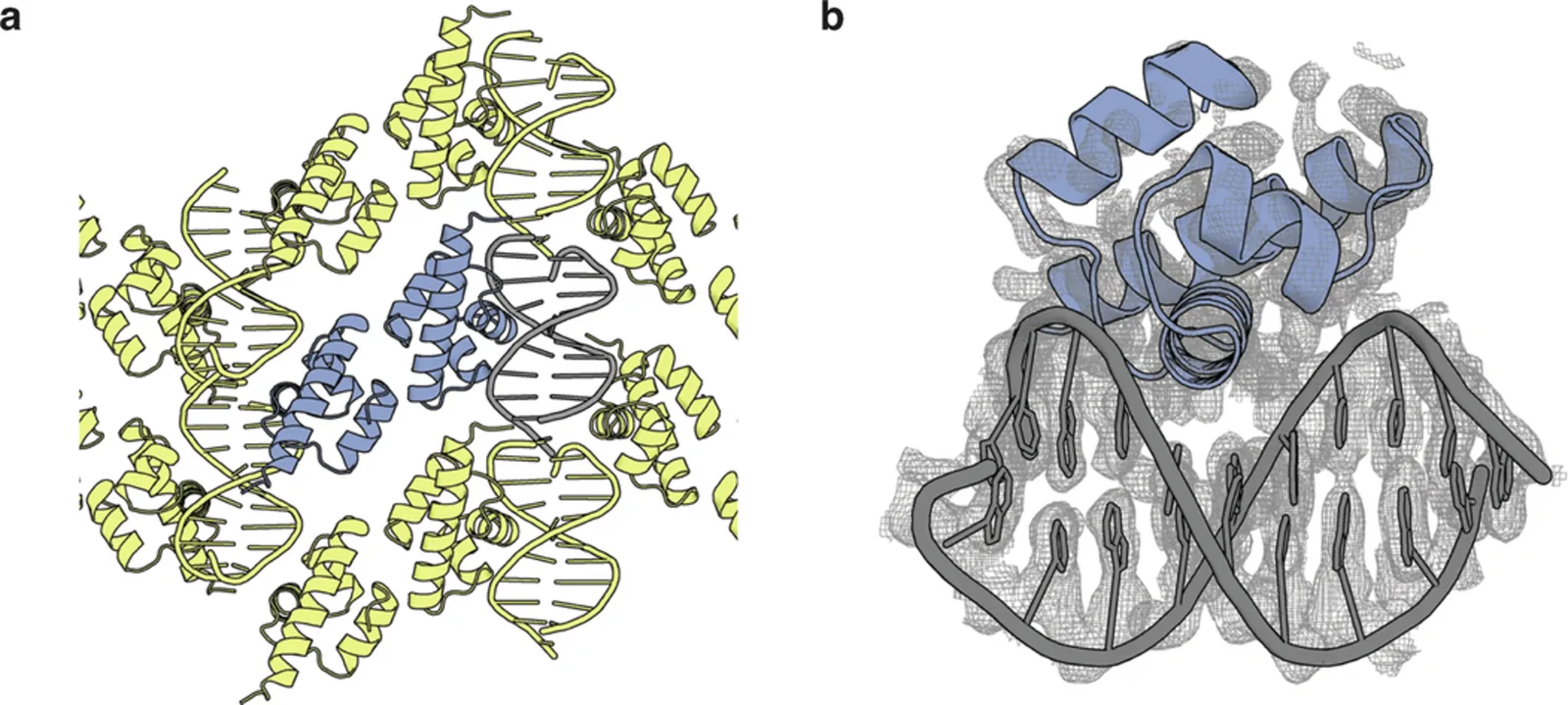
Global view of the DBP48 co-crystal structure. **a**, Packing of the DBP48 co-crystal structure with asymmetric unit highlighted in blue. **b**, Global density of the DBP48 crystal structure.

We solved the cocrystal structure of DBP 48 in complex with its preferred target sequence and found very close agreement to the design model (Table 1, Fig. 3a and Extended Data Fig. 7). Cα root-mean-square deviation (r.m.s.d.) of the cocrystal structure to the design model was 0.64 Å for the binder alone and all-atom r.m.s.d. was 1.907 Å for the protein–DNA complex. Among interface residues forming key interactions with bases, R38 and S39 were in the closest agreement and formed the expected side chain–base hydrogen bonds (Fig. 3b). D43 and R49 did not form the expected hydrogen bonds observed in the design model, likely because of slight differences in orientation of the binder to DNA and deviations from ideal B-DNA in the cocrystal structure. D43 was instead involved in a water-mediated hydrogen bond to C11 (Fig. 3c) and R49 was part of a hydrogen-bond network involving the phosphate backbone. An additional water-mediated hydrogen bond was observed between S42 and A10. While water-mediated interactions are not considered by the Rosetta protocol used to build the side chains in the final design model, the LigandMPNN sequence design method implicitly considers these as the PDB training set contains many examples of water-mediated hydrogen bonds, which are known to confer additional specificity in native DBPs^20,21,37^. Extensive hydrogen-bond networks were also observed with the DNA phosphate backbone, with most involved protein residues supported by side chain–side chain hydrogen bonds and packing interactions. These hydrogen-bond networks with the phosphate backbone imply that much of the docking orientation is dominated by these interactions, suggesting that further enrichment for these features could improve design success rates.

To assess the contributions of each amino acid to binding for additional designs, we generated high-resolution footprints of the binding surface by sorting site saturation mutagenesis (SSM) libraries in which every residue was substituted with each of the 20 amino acids one at a time for DBPs 1, 6 and 35 (Fig. 3d,e and Supplementary Figs. 5 and 6). For each of the three designs, we found that most positions at the interface and the core were largely conserved, while positions at the surface were more tolerant of substitutions. In a small number of cases, substitutions led to notable improvements in binding affinity. The depletion of most substitutions in both the binding site and the core suggests that the design models are largely correct, whereas the enriched substitutions suggest routes to improving affinity.

### Assessment and optimization of designed DBP specificity

**Fig. 4:**
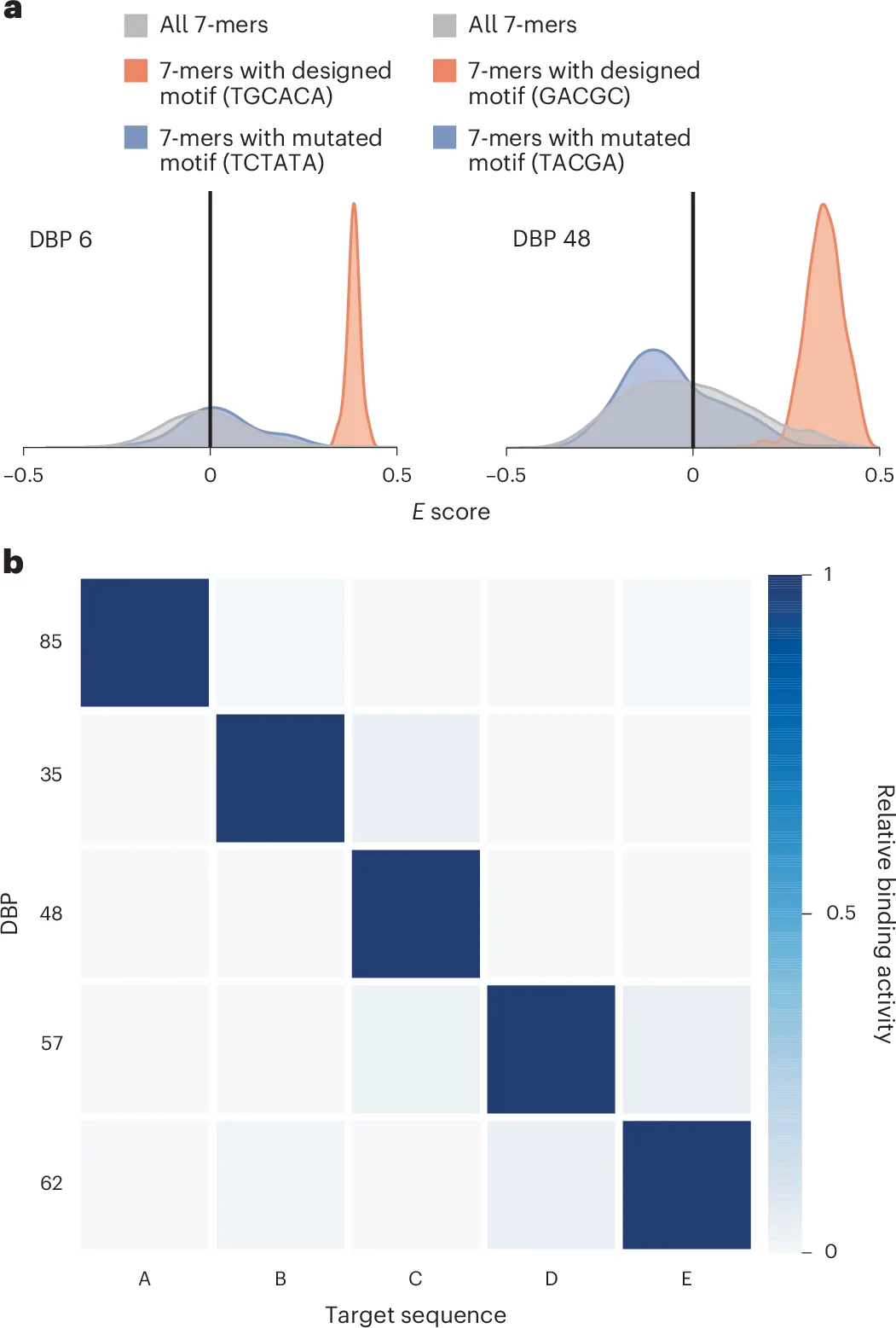
Designed DBPs are highly specific. **a**, Histograms of *E*-score values for DBP 6 (top) and DBP 48 (bottom) from uPBMs showing high specificity to the designed target sequence. The *E*-score distribution of all 7-mers is shown in gray, the distribution of 7-mers containing the designed binding-site motif is shown in orange and the distribution for a mutated binding-site motif is shown in blue. **b**, All-by-all orthogonality matrix for five designed DNA binders screened by yeast display, normalized by row, at a DNA concentration of 1 µM (with avidity). Full orthogonality matrix with all tested DNA targets shown in Extended Data Fig. 9i. Source data

**Extended Data Fig. 8:**
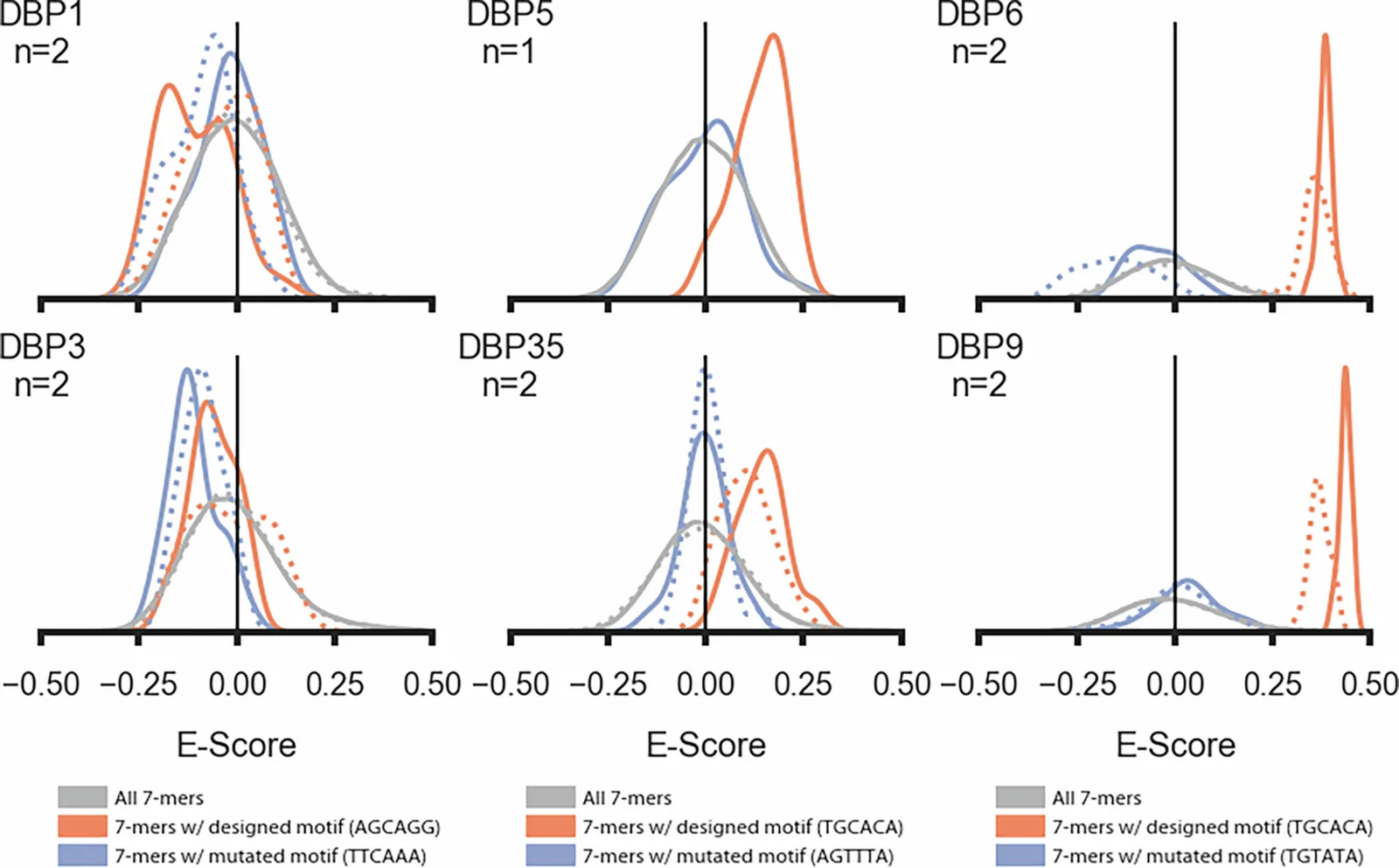
Analysis of DBPs 1, 3, 5, 6, 9, 48, and 35 with universal protein binding microarray experiments containing all 7-mers. Solid lines represent replicate 1 while dashed lines represent replicate 2, where applicable. DBPs 6, 9, and 48 were highly specific to the intended target and the mean percentile rank of 7-mers containing the designed binding site 5-mer or 6-mer was 99.54%, 99.89%, and 97.59%, respectively. DBPs 5 and 35 were less specific to their target site but still preferred the target motif over sequences with a mutated binding motif (designed motif percentile 86.54% and 81.88%, respectively). DBPs 1 and 3 did not appear to have a preference to the designed target site (33.19% and 46.58%, respectively). Source data

**Extended Data Fig. 9:**
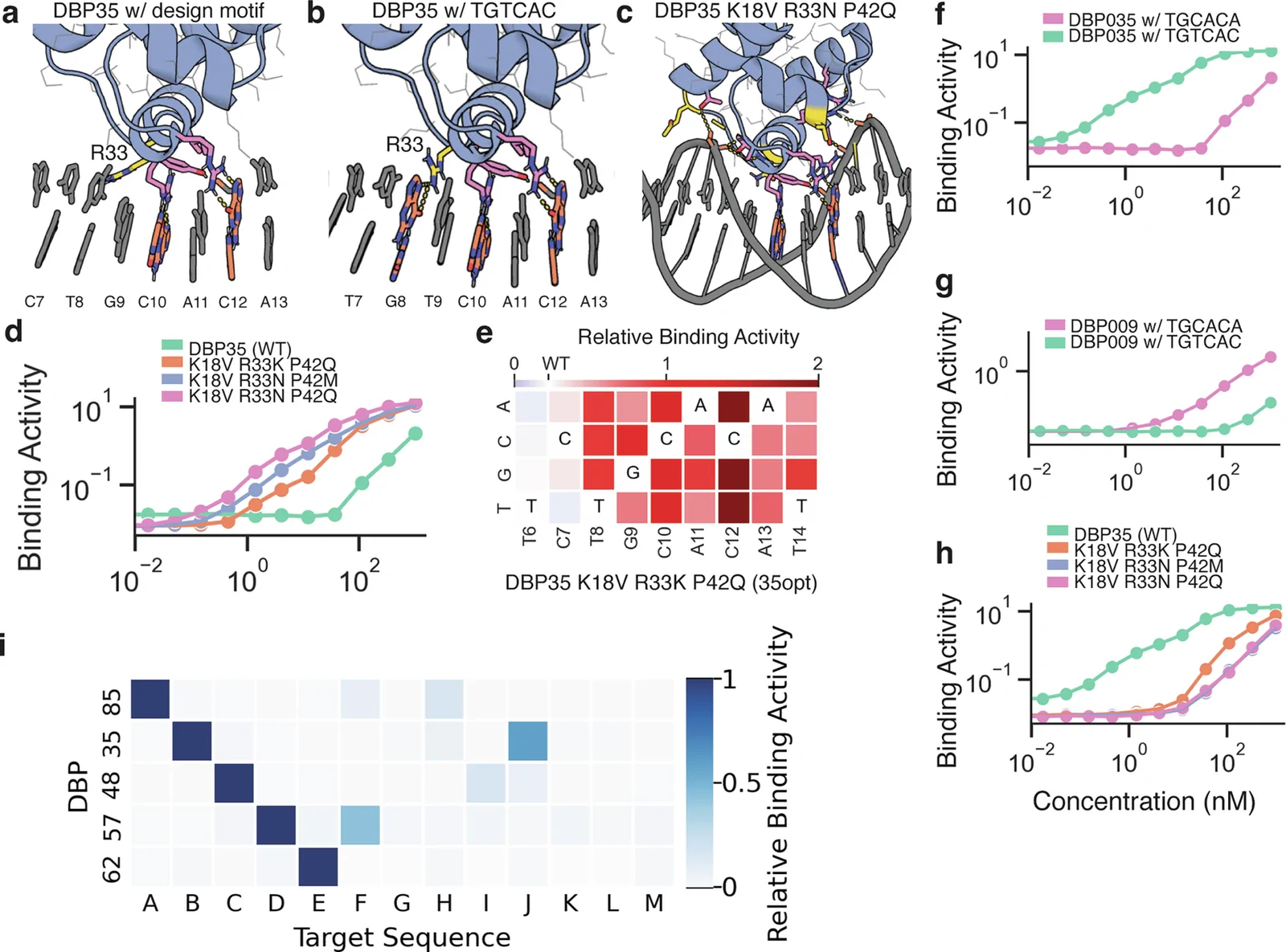
Optimizing DBP35 to disrupt off-target DNA binding. 7-mers containing TGTCAC were enriched in designs targeting the sequence B (1L3L) dsDNA oligo in uPBM experiments. **a**, Design structure of DBP35 with R33 highlighted in yellow. **b**, Structure of DBP35 modeled with 7-mer TGTCACA shows R33 forming a potential hydrogen bond with G8. **c**, Structure of DBP35 modeled with affinity enhancing mutations K18V, R33N, and P42Q informed by SSM experiments. **d**, Binding activity (PE/FITC) from yeast display titration (without avidity) of biotinylated dsDNA target shows several orders of magnitude improvement in binding activity in DBP35 combo mutants, with binding signal detectable with dsDNA labeling below 1 nM. **e**, Relative binding activity (Normalized PE/FITC) from a yeast display competition assay of DBP35 K18V R33N P42Q showing substantial improvement in specificity over DBP35 (Fig. 2C). Competition assay was performed with biotinylated dsDNA target at 20 nM and competitor dsDNA at 160 nM. **f**, Yeast display titration (without avidity) showing binding activity (Median PE/FITC) of DBP35 with biotinylated dsDNA target containing designed target motif (CTGCACA) or substituted with alternative target motif (TGTCACA) shows increase in binding strength for TGTCACA over CTGCACA. **g**, Yeast display titration (without avidity) of wildtype DBP9 shows that the designed target motif is strongly preferred over the off-target sequence. **h**, Combo mutants of DBP35 show significant disruption of binding to dsDNA oligos containing the alternate TGTCAC motif by yeast display. **i**, Orthogonality matrix for 5 designed DNA binders screened by yeast display against all target sequences for which designs were made, normalized by row, at a DNA concentration of 1uM (with avidity). Source data

We selected seven designs for further assessment of specificity in vitro using universal protein-binding microarrays (uPBMs) containing all possible 7-mer DNA sequences^38,39^. We found that several designs exhibited very high specificity for their design targets, most notably DBPs 6, 9 and 48, for which 7-mers containing the binding-site motif were highly preferred over those lacking the motif (Fig. 4a and Extended Data Fig. 8). All-by-all analysis of on-target DBPs (Extended Data Fig. 2) showed that, while some designs exhibited off-target binding, a number were highly specific to a single target. Five of our designed DBP–target pairs were highly orthogonal (Fig. 4b and Extended Data Fig. 9i).

For DBP 35, which had moderate preference for the designed motif (Extended Data Fig. 8), we explored optimization of the specificity and affinity by combining substitutions found in mutational scanning. We found that combining three mutations into an optimized variant (DBP 35opt) (Extended Data Fig. 9a–c) (R33N, which prevents a potential off-target interaction; K18V, which adds an additional hydrophobic interaction with the methyl stem of base ADE11; P42Q, which potentially stabilizes the protein scaffold structure) dramatically increased binding strength observed by yeast display with detectable binding down to ~150 pM (Extended Data Fig. 9d). These mutations also increased specificity to seven base-pair positions as observed in a yeast competition assay (Extended Data Fig. 9e) compared to the specificity to three base-pair positions observed in the original design (Fig. 2b) and substantially reduced binding to off-target motifs (Supplementary Fig. 1f–h). Thus, just a few mutations of an initial design can lead to dramatic improvements in specificity and affinity.

### Designed DBPs modulate transcription in living cells

**Fig. 5:**
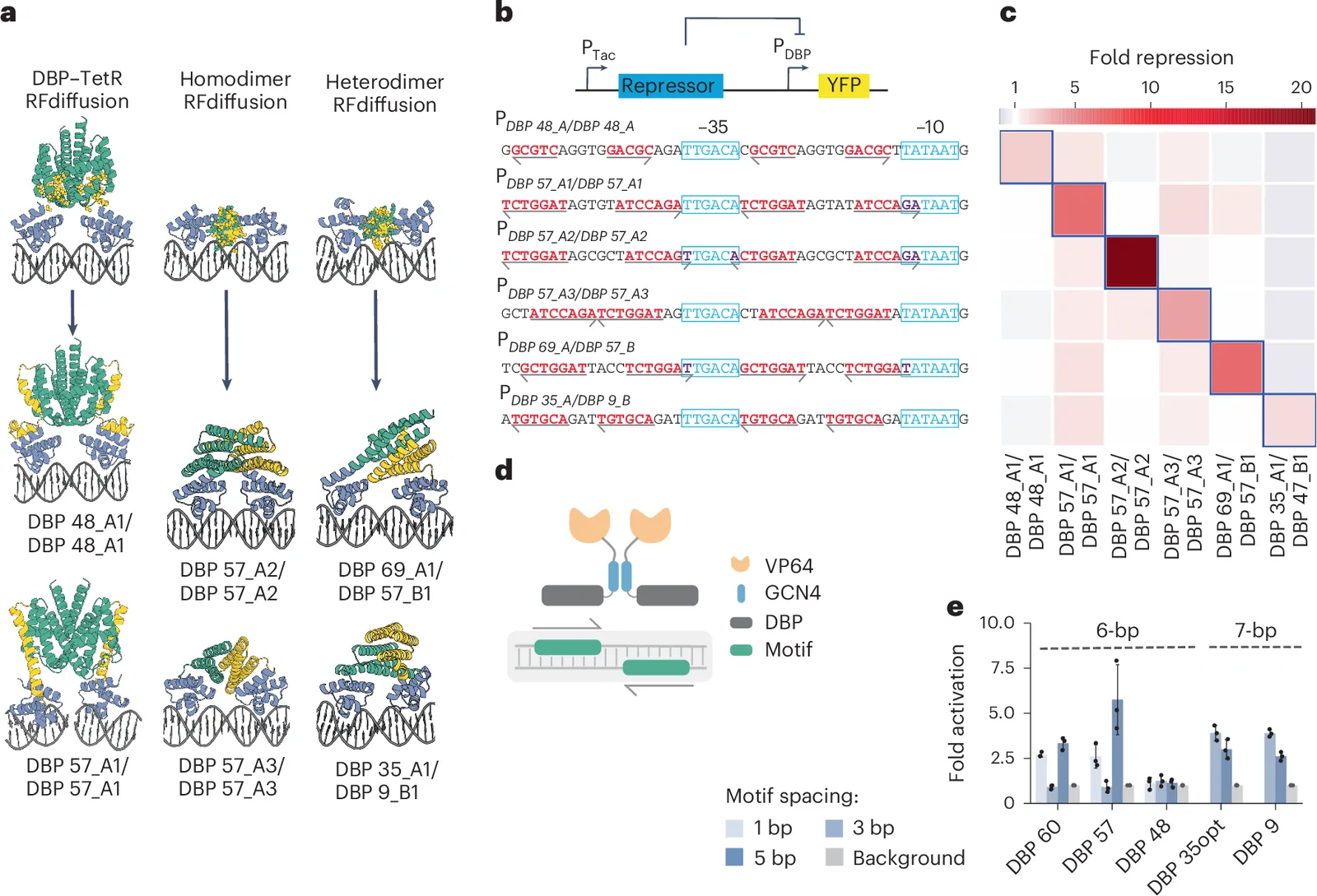
Designed DBPs function in living cells to direct transcriptional repression and activation. **a**, Illustration of the RFdiffusion method for building out DBP domains into homodimer or heterodimer arrangements, along with repressor designs selected for all-by-all repression assays. DBP 48_A1/DBP 48_A1 and DBP 57_A1/DBP 57_A1 are homodimer constructs transitioned into the TetR backbone while the remainder are de novo homodimer or heterodimer constructs. **b**, Transcriptional repression in *E.* *coli*. Functional IPTG-inducible repressor block transcription of YFP from a synthetic promoter containing the designed DBP DNA-binding sites (red text) around the −10 and −35 elements (blue text). Arrows indicate directionality of the binding site. **c**, All-by-all orthogonality matrix showing fold repression of YFP fluorescence from flow cytometry analysis of cells containing the successful NOT gate circuits. Blue outlines indicate on-target repressor–promoter pairs. **d**, Transcriptional activation in HEK293T cells measured by ENGRAM. synTFs were created by fusing the GCN4 dimerization domain and the VP64 activation domain to the C termini of the DBPs. The synTF-specific CREs were created by evenly distributing palindromic binding motifs on a 130-bp transcriptionally inactive DNA sequence where each CRE drives a uniquely barcoded pegRNA for recording into DNA TAPE. **e**, Fold activation of synTFs measured as normalized barcode abundance. Dots represent individual data points, bars represent mean fold activation and error bars represent the s.d. of the mean relative barcode abundance (*n* = 3 biological replicates). Source data

**Extended Data Fig. 10:**
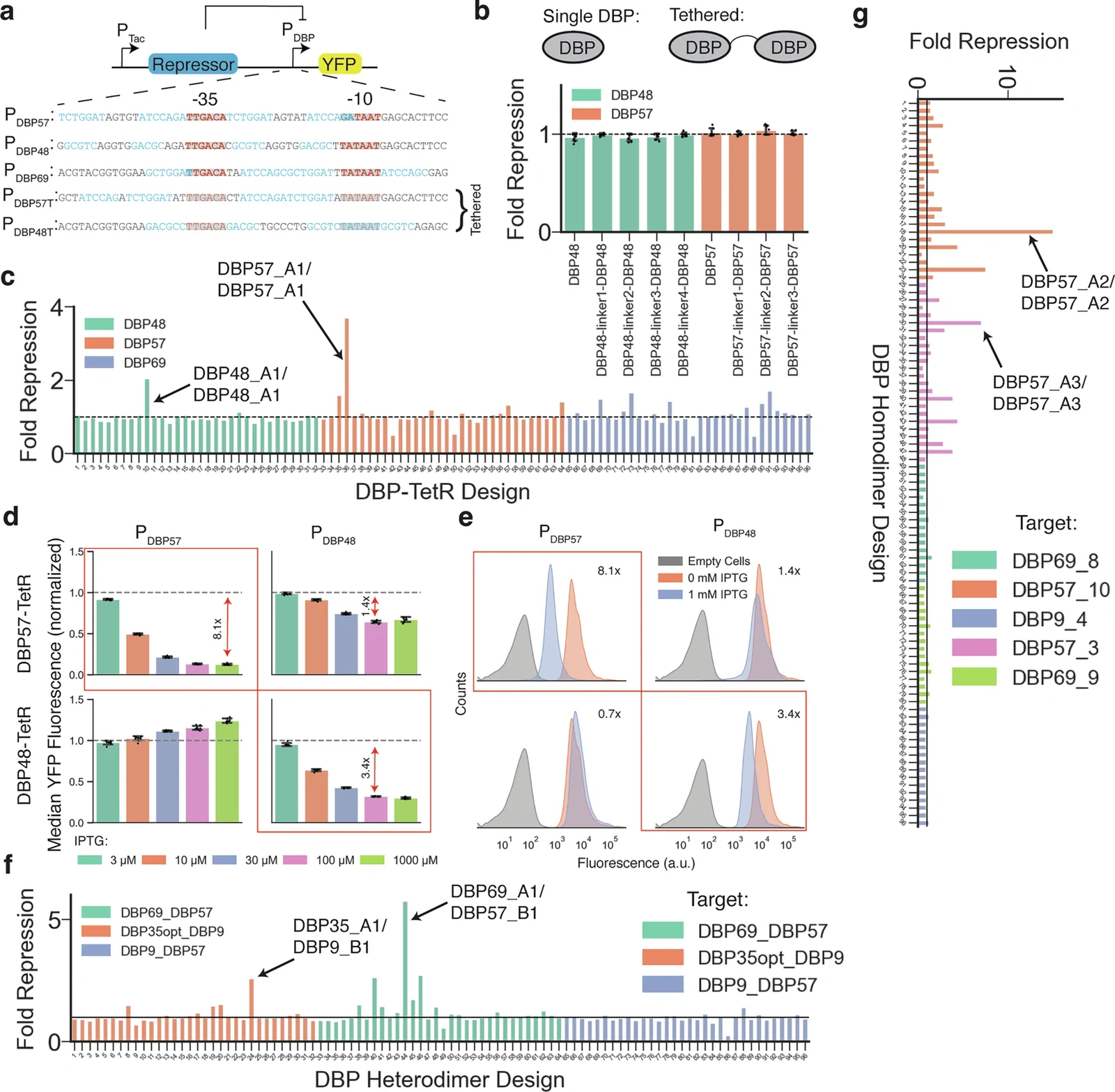
Use of DBPs to direct transcriptional repression in *E. coli*. ***a***, Vectors encoding the repressor variants were constructed with a repressor under control of the IPTG-inducible P_Tac_ promoter. A synthetic promoter containing the designed DBP binding sites (blue text) around the -10 and -35 elements (red text) was used to control expression of YFP. **b**, Fold repression was not observed at 1 mM IPTG induction as determined by flow cytometry analysis of cells containing single DBP domains (DBP57, DBP48) and tandem linked DBP domains used as repressors. Error bars represent standard error of the mean of n = 4 biological replicates. **c**, Fold repression of 96 DBP-TetR designs revealed substantial repression for at least two variants incorporating DBP57 and DBP48 in cells induced at 0.1 mM IPTG. n = 1. **d**, Normalized median YFP Fluorescence from flow cytometry analysis of cells containing the successful DBP57-TetR (upper) and DBP48-TetR (lower) NOT gate circuits. Error bars represent standard deviation of the mean (n = 7 biological replicates represented as dots). **e**, Representative histograms of YFP fluorescence from *E. coli* cells transformed with DBP-TetR NOT circuits. Fold repression of YFP was ~8.1x and ~3.4x for DBP57-TetR (upper left) and DBP48-TetR (lower right) repressor variants, respectively, when encoded with their cognate promoters upon induction with 1 mM IPTG. Fold repression is indicated in each subplot. Error bars represent standard error of the mean of n = 8 biological replicates. **f-g**, Fold repression of 96 DBP heterodimer (**f)** and homodimer (**g**) designs revealed substantial repression for 4 and 7 variants, respectively, in cells induced at 1 mM IPTG. Source data

We tested the ability of our designed DBPs to function in cells to regulate transcription. To assay transcriptional repression in *E.* *coli*, we constructed candidate NOT gates^40^, where the input is a designed DBP under control of the IPTG-inducible P_Tac_ promoter and the output is yellow fluorescent protein (YFP) expression driven by a promoter incorporating the DBP DNA-binding site (Extended Data Fig. 10a). Single DBP domains and two copies of the same DBPs tethered through a flexible linker failed to exhibit YFP repression upon IPTG induction (Extended Data Fig. 10b), suggesting a need for higher-affinity binding, longer sequence recognition and/or a bulkier binding protein for effective hindrance of transcription initiation by *E.* *coli* RNA polymerase. To increase avidity and bulk, we positioned two copies of the same DBP (or one copy each of two different DBPs) on B-form DNA containing two palindromic copies of the target site (or the two different target sites), separated by different numbers of bases. We then used RFdiffusion^41^ to build out new protein backbone segments that either transition into the TetR homodimer^42^ or interact directly in homodimeric or heterodimeric arrangements (Fig. 5a). Following sequence design with ProteinMPNN to favor folding and assembly of the extensions to the intended dimeric structure, we used AF2 to predict the structures of the homodimers and heterodimers and selected those that were close to the design models. We experimentally characterized the ability of these designs to repress transcription from synthetic promoters incorporating two dimer binding sites (four individual domain-binding sites in total) flanking the −35 promoter region. Dose-dependent repression (>2-fold) was observed for 2 of 96 TetR-incorporating homodimeric designs (Extended Data Fig. 10c–e) and 18 of 192 entirely de novo homodimeric and heterodimeric repressors incorporating different designed DBPs (Fig. 5a and Extended Data Fig. 10f,g). All-by-all characterization of six selected designs and the corresponding cognate promoters showed considerable orthogonality (Fig. 5b,c), with up to 20-fold repression for cells with the cognate target (DBP 57_A2/DBP 57_A2). Notably, two de novo dimeric designs with DBP 57 designed to bind palindromic arrangements of the cognate target site at different spacings and in different orientations were each specific for their intended target, indicating that a single domain can serve as the basis for creating an array of orthogonal repressors.

Next, we tested DBP function as activators in mammalian cells. A set of synthetic transcription factors (synTFs) were created by fusing the GCN4 dimerization domain and the VP64 activation domain to the C termini of DBPs 9, 35opt, 48, 57 and 60, which collectively recognize three unique motifs. The dimerization domain allows the DBPs to recognize a palindromic target sequence consisting of two binding motifs, increasing the binding affinity to the DNA sequence. We used the ENGRAM^43^ recording technology to measure the activity of specific *cis*-regulatory elements (CREs) in HEK293 cells (Fig. 5d). In ENGRAM, each CRE drives expression of a uniquely barcoded pegRNA, which, upon expression, is recorded into the DNA TAPE at the HEK3 locus by prime editor PEMax. After analyzing the barcode abundance for each individual CRE, we observed 3–5-fold activation for DBPs 9, 35opt, 57 and 60 (Fig. 5e).

### Determinants of DBP design success

Across all targets, designs that bound specifically to their intended target (Extended Data Fig. 1) tended to have more side-chain and main-chain phosphate hydrogen bonds, lower Rosetta *ΔΔG* and lower Cα r.m.s.d. of the AF2-predicted structure to the design model (Supplementary Fig. 7), while nonspecific binding was strongly correlated with a positive net charge. We did not observe enrichment of higher RotamerBoltzmann probabilities for side chains that make hydrogen bonds with bases, likely because of prior enrichment in the ordered design sets. However, we did observe enrichment of higher RotamerBoltzmann probabilities for side chains that make hydrogen bonds with phosphates (Supplementary Fig. 7). Further enrichment for these metrics should increase design success rates. For the DNA sequence targets where we generated successful binder hits and used the same design procedure against both ideal B-DNA and DNA derived from a cocrystal structure (design set 2), we did not observe a notable difference in success rates (eight hits with B-DNA and six hits with cocrystal-derived DNA).

A key feature of our design method is sampling from numerous diverse starting structures and docking positions to find complexes that can engage both the bases for sequence-specific recognition and the phosphate backbone to favor the designed binding mode. Like the most specific of our designs, native DBPs also have geometries enabling formation of main-chain phosphate hydrogen bonds (Supplementary Fig. 8) and highly preorganized side-chain phosphate hydrogen bonds (Supplementary Fig. 2). This is perhaps because of the inherent rigidity of these interactions that favor specific docks and restrict otherwise possible interactions of flexible side chains with off-target DNA base atoms. To explore the importance of phosphate contacts mediating specific docks for achieving specificity to a given target site, we performed LigandMPNN redesign of 14 hits from our design campaigns against 100 randomly generated target sequences. Upon Rosetta relaxation of the redesigned complexes (20 LigandMPNN designed proteins per target–scaffold pair) in the presence of DNA, we observed that only two of the 100 sequences have as favorable Rosetta *ΔΔG* values and as many hydrogen bonds to bases (Supplementary Fig. 9), suggesting that the details of the scaffold backbone and dock make important indirect contributions to specificity by locking in the exact binding mode and narrowing the range of possible side chain–base contacts. This makes it difficult to design DBPs to new DNA sequences through a native redesign approach and highlights the advantage of our computational sampling-based approach.

## Discussion

Our computational DNA binder design approach can generate DBPs that specifically bind arbitrary DNA sequences, including sequences that are not bound by known DBPs in the PDB or JASPAR databases. These designed DBPs function both in vitro and in living cells, as assessed through transcriptional repression and activation assays in both *E.* *coli* and eukaryotic cells, respectively. The method samples structurally diverse HTH scaffolds to identify complexes that can facilitate specific contacts with the target DNA bases. The best designs were highly specific for their intended targets and the crystal structure and specificity profiling assays strongly corroborate the computational design models. The modularity of the binding domains enables further increases in specificity by rigidly positioning multiple modules along the DNA double helix using RFdiffusion; as illustrated in Fig. 5b, two designs having two copies of the same binding module at different spacings each modulate transcription at a tandem target site with matching spacing but not at a target site with mismatched spacing.

The design method presented in this paper now enables the design of custom DNA-binding miniproteins to target specific DNA sequences for diverse applications in gene regulation and editing. While we focused on design of HTH domains as a proof of principle, the method should be extensible to DBP families beyond the HTH domains used here, particularly those using a helix in the major groove for recognition such Cys_2_His_2_ ZF domains or homeodomains, by generating scaffolds models with structure prediction tools; using scaffolds containing extensive β-sheets or loops, such as p53-like TFs^44^ could be more difficult because of their increased flexibility but should still be feasible. Beyond extending to a broader range of scaffolds, future design improvements would include consideration of sequence-dependent DNA shape, induced fit and the role of water-mediated hydrogen bonds, all of which are known to have an important role in sequence recognition^25,26,45^. We focused on design of direct side chain-base interactions against a fixed target DNA sequence for simplicity and because of the accuracy and computational costs of existing tools to model DNA conformational flexibility and water-mediated interactions. Moving forward, it should become possible to model these features more accurately and more computationally-efficiently using machine learning methods such as existing or future variants of RoseTTAFold-NA^46^, AF3 (ref. ^47^) and RFdiffusion-allatom^48^.

The ability to incorporate designed DBPs into transcriptional regulators through homodimerization and heterodimerization (Fig. 5) should allow the expansion of orthogonal TF–operator pairs for more complex gene circuits^49^. As outlined in Fig. 5, using RFdiffusion, it should be straightforward to fuse DNA-binding miniproteins together in a single chain in defined spatial orientations to allow specific targeting of longer target sites or link DBPs with epigenetic modifiers or other effector recruiting domains to provide functionality beyond transcriptional activation and repression. Computationally designed DBPs are also well suited for the simultaneous recognition of both DNA sequence and shape, including non-B-DNA structures that may occur in ~13% of the human genome^50^. For any given backbone, there will likely be limitations in the ability to target certain DNA sequences, which likely explains the challenges in generating Cys_2_His_2_ ZF and other native scaffolds to bind to some target sites; our approach provides a powerful new method for building DBPs with backbones tailored to specific sequences of interest and we anticipate that the method and the sequence-specific designs should be widely useful in synthetic biology and other areas requiring sequence-specific DNA recognition.

## Methods

### Scaffold library generation

Scaffolds deposited to the PDB with structural similarity to selected template backbones (PDB 1L3L (ref. ^51^) and PDB 1PER (ref. ^52^)) were identified using TM-align^29^. Amino acid sequences of identified protein scaffolds were used as seeds to generate multiple-sequence alignments (MSAs) using an HHBlits^53^ search of the UniRef30 database^54^. Resulting MSAs were used for HMMer^55^ searches of the JGI metagenome protein sequence databases^56^ and the Uniref100 database^54^. HMMer search results were clustered to <70% sequence identity using MMSeqs2 (ref. ^57^) and MSAs were generated from each clustered sequence using HHBlits. AF2 (ref. ^28^) was used to predict structures for each sequence using the generated MSAs. Resulting scaffolds were filtered for high confidence AF2 pLDDT scores, TMscore to the input backbone templates and Rosetta score. Scaffolds of specific topologies were supplemented with additional AF2-predicted structures of TF sequences identified from bacterial metagenomes using DeepTF^58^. PSSMs were generated for each scaffold using PSI-Blast^59^ and custom code for use as constraints of Rosetta design. All final scaffolds are available for download.

### RIF docking of scaffolds onto DNA targets (DBP design step 1)

Structures of B-DNA for each target (Supplementary Table 2) were generated by (1) using the DNA portion of PDB 1BC8 (ref. ^60^), PDB 1YO5 (ref. ^61^), PDB 1L3L (ref. ^51^) or PDB 2O4A (ref. ^62^) or (2) using the software X3DNA^63^, followed by a constrained Rosetta relax of the DNA structure. RIFdock was allowed to target along the entire stretch of each target sequence. The RIF docking method performs a high-resolution search of continuous rigid-body docking space. RIF docking comprises two steps. In the first step, ensembles of interacting discrete side chains (referred to as ‘rotamers’) tailored to the target are generated. Polar rotamers are placed on the basis of hydrogen-bond geometry, whereas apolar rotamers are generated through a docking process and filtered by an energy threshold. Rotamers were only calculated for nucleotide base atoms in the major groove of the DNA target. All the RIF rotamers are stored in ~0.5-Å sparse binning of the six-dimensional rigid-body space of their backbones, allowing extremely rapid lookup of rotamers that align with a given scaffold position. To enrich for canonical protein–DNA hydrogen-bond interactions, rotamers of arginine, glutamine and asparagine forming bidentate hydrogen bonds with G and A bases were extracted from the PDB, clustered by r.m.s.d., aligned to the DNA target at all G and A positions and added to the RIF as hotspot residues. To facilitate the next docking step, RIF rotamers are further binned at 1.0-Å, 2.0-Å, 4.0-Å, 8.0-Å and 16.0-Å resolution. In the second step, a set of scaffolds was docked into the produced rotamer ensembles, using a hierarchical branch-and-bound search strategy. Starting with the coarsest 16.0-Å resolution, an enumerative search of scaffold positions was performed; the designable scaffold backbone positions were checked against the RIF to determine whether rotamers could be placed with favorable interacting scores. All acceptable scaffold positions (up to a configurable limit, typically 10 million) were ranked and promoted to the next search stage. Each promoted scaffold was split into 26 child positions in the six-dimensional rigid-body space, providing a finer sampling. The search was iterated at 8.0-Å, 4.0-Å, 2.0-Å, 1.0-Å and 0.5-Å resolutions. All RIF docks were required to use at least one hotspot residue to be saved as an output.

### Energy function optimization

The next steps of the DBP design pipeline after RIFdock involved sequence design and/or modeling protocols with Rosetta. To facilitate this, a new version of the Rosetta score function was trained to better evaluate the energy of protein–DNA interfaces. Additional flexibility of the DNA duplex was incorporated into Rosetta’s rotamer optimization and gradient-based minimization modules using modifications of DNA dihedral angles^64^ and the score function was optimized using the same general method as previously published^65^. The weights of individual terms in the score function were optimized to reproduce the geometries of DNA crystal structures. Specifically, the distributions of pairwise atomic distances, base-stacking and base-pairing geometries and bond torsions were considered. Additional optimization was performed on tasks related to protein–DNA complex structures. These tasks included energy ranking of perturbed crystal structures, rotamer recovery in repacking crystal structures and sequence recovery in redesigning the protein sequence of crystal structures. An additional weight was placed on the frequency of positively charged residues at interface positions because previous score functions tended to overestimate the strength of solvent-exposed charged interactions. Similar geometric and design tasks were included for protein structures alone. Rosetta score weights optimized included partial atomic charges of protein and DNA, hydrogen-bond strengths and solvation energies. The resulting score function showed improvement across nearly all tasks, with the greatest improvements found in the protein–DNA energy ranking and sequence design.

### Rosetta-based interface sequence design (DBP design step 2, option A)

A stripped down version of the Rosetta score function was used to roughly design the interface of RIF dock outputs^6^. This step was primarily used to replace clashing residues before evaluating for design potential. Specifically, fa_elec, lk_ball[iso,bridge,bridge_unclp] and the _intra_ terms were disabled. All that remained were Lennard–Jones, implicit solvation and backbone-dependent one-body energies (fa_dun, p_aa_pp and rama_prepro). Additionally, flags were used to limit the number of rotamers built at each position (Supplementary Information). After the rapid design step, the designs were minimized twice: once with a low-repulsive score function and again with a normal-repulsive score function. Rosetta *ΔΔG* and contact molecular surface were then calculated on the roughly designed interface. A maximum-likelihood estimator was used to give each predicted design a likelihood that it should be selected to move forward. A subset of the docks to be evaluated were subjected to the full sequence design and their final metric values were calculated. With a goal threshold for each filter, each fully designed output can be marked as pass or fail for each metric independently. Then, by binning the fully designed outputs by their values from the rapid trajectory and plotting the fraction of designs that pass the goal threshold, the probability that each predicted design passes each filter can be calculated. From here, the probability of passing each filter may be multiplied together to arrive at the final probability of passing all filters. This final probability can then be used to rank the designs and pick the best designs to move forward to full sequence optimization. Note that the rapid design protocol here is used merely to rank the designs, not to optimize them; the original docks are the structures carried forward.

These docked conformations passing the rapid design protocol were further optimized to generate shape-complementary and chemically complementary interfaces using a Rosetta FastDesign protocol, alternating between side-chain rotamer optimization and gradient-descent-based energy minimization. Design was performed with a sequence profile constraint based on an MSA of the originating native scaffold sequence and cross-interface interactions upweighted to maximize contacts and shape complementarity. We did not allow Rosetta to repack or relax the DNA target during the design procedure. A Python script was implemented to automatically carry out rapid design evaluation, preemption and full sequence design. Computational metrics of the final design models were calculated using Rosetta, which includes *ΔΔG*, hydrogen bonds to base atoms and contact molecular surface, among others, for design selection. All the script and flag files to run the programs are provided in the Supplementary Information. ProteinMPNN was used to redesign noninterface residues in the final design step, before AF2 monomer validation.

### LigandMPNN-based sequence design (DBP design step 2, option B)

LigandMPNN was used for sequence design in the context of DNA. The network was used to optimize the protein sequence for given protein–DNA complex structures during design, whereby amino acids were determined autoregressively by the identity and location of neighboring protein and DNA residues. When the full protein sequence was determined, it was threaded onto the input protein scaffold. As in the above Rosetta-based interface sequence design protocol, the designs were minimized with a low-repulsive score function and again with a normal-repulsive score function and Rosetta *ΔΔG* and contact molecular surface were calculated on the roughly designed interface. A maximum-likelihood estimator was used to pre-empt design of poor docks as described in the above Rosetta-based sequence design protocol. A Python script was implemented to automatically carry out MPNN sequence design, rapid design evaluation, preemption and Rosetta Relax. Computational metrics of the final design models were calculated using Rosetta, which includes *ΔΔG*, interface hydrogen bonds and contact molecular surface, among others. LigandMPNN temperatures of 0.2–0.3 were used earlier in the design process to increase the variability of amino acid sequences, while a temperature of 0.1 was used later to determine the more probable sequences. Key residues making base-specific hydrogen bonds with DNA atoms were fixed in later stages of the pipeline to encourage the design of supporting residues. All the script and flag files to run the programs are provided in the Supplementary Information.

### Backbone resampling with motif grafting (DBP design step 3, option A)

Motif grafting was performed as previously reported^6^. Briefly, the binding energy and interface metrics for all the continuous secondary structure motifs (helix, strand and loop) were calculated for the designs generated in the broad search stage, as performed in previous work^6^. The motifs with good interactions (based on binding energy and other interface metrics, such as contact molecular surface) with the target were extracted and aligned using the target structure as the reference. All the motifs were then clustered on the basis of an energy-based TM-align-like clustering algorithm^29^ without any further superimposition. The best motif from each cluster was then selected on the basis of the per-position weighted Rosetta binding energy, using the average energy across all the aligned motifs at each position as the weight. Around 500–2,000 best motifs were selected and the scaffold library was superimposed onto these motifs using the MotifGraft mover^66^. Interface sequences were further optimized and computational metrics were computed for the final optimized designs as described in the Rosetta-based and LigandMPNN-based sequence design methods.

### Backbone remodeling with protein inpainting (DBP design step 3, option B)

Scaffold secondary structures were determined using DSSP^67^. ProteinInpainting contigs were generated for each design that mask scaffold loops longer than four residues and surrounding residues while ensuring that all residues forming hydrogen bonds to the DNA backbone were conserved. In total, 10–20 unique contigs were generated for each design and sequences were constrained to a maximum of 65 aa. ProteinInpainting outputs were aligned to the DNA target using fixed interface residues of the input structure. The aligned ProteinInpainting outputs were subject to several further LigandMPNN + FastRelax rounds (DBP design steps 4 and 5) before AF2 monomer prediction and superposition steps.

### AF2 monomer validation and superposition (DBP design step 5)

AF2 structures were produced using the single sequence of each design. AF2 was run with model 1 and 12 recycles for each design. The Cα r.m.s.d. of the AF2 structures to each respective design model was calculated. AF2 structures were superpositioned onto the DNA target using the backbone coordinates of interface residues within 8 Å of the DNA target. A fixed backbone Rosetta FastRelax was performed on each superpositioned complex and all relevant metrics were calculated on the final superpositioned design model.

### Design filtering (DBP design step 6)

Designs were filtered after each sequence design step and after superimposition of AF2 models for those with the most favorable free energy of binding (Rosetta *ΔΔG*), contact molecular surface area^6^ and interface hydrogen bonds, the fewest interface buried unsatisfied hydrogen-bond donors and acceptors and those containing bidentate side chain–base hydrogen-bonding arrangements frequent in the PDB, including bidentate interactions of R–G, Q–A and N–A. Designs were additionally filtered for those with a high RotamerBoltzmann score (see below) among arginine, lysine, glutamine or asparagine residues forming hydrogen bonds with bases (max rboltz RKQE) and those with a high median RotamerBoltzmann (median rboltz) score of all residues forming hydrogen bonds with bases.

### RotamerBoltzmann filters

The Boltzmann probability of finding a given rotamer in a specific state was evaluated using the RotamerBoltzmannWeight filter in Rosetta^32^. The RotamerBoltzmann score is an approximation of preorganization of a given residue in the unbound state. All amino acid residues forming hydrogen bonds with DNA base or phosphate atoms were evaluated by this metric, which was calculated on the protein monomer in the unbound state. The metric was estimated by fixing neighboring side chains and assessing the Boltzmann probability distribution on rotamers accessible by the side chain of interest. To increase the likelihood of a given rotamer in the protein–DNA complex, designs with lower RotamerBoltzmann scores (a score of 0 implies the rotameric state is unpopulated and a score of 1 implies the state is the only populated state) were preferentially chosen, as known native protein–DNA crystal structures tend to contain preorganized amino acid residues (Supplementary Fig. 2).

### Analysis of design from native cocomplexes

To examine the ability of LigandMPNN-based sequence design to generate interfaces passing our in silico metrics when starting from crystal structures of native cocomplexes, we identified cocomplexes from the PDB with high TM-align to the designed DBPs. We mutated the DNA sequence in silico to the target sequence. In cases where the register of the DNA in the crystal structure complex did not match the design model, we systematically slid the design motif sequence, exploring all possible offsets and generating rethreaded structures for each sequence alignment. We used LigandMPNN to redesign the entire protein sequence of each native complex followed by side-chain relaxation using Rosetta FastRelax. To assess the resemblance between redesigned natives and designed DBP motifs, we examined whether the same amino acids formed hydrogen bonds with the same DNA base atoms (motif interaction recovery).

### DNA library preparation

All protein sequences were padded to 65 aa by adding a (GGS)_*n*_ linker at the C terminus of the designs to avoid the biased amplification of short DNA fragments during PCR reactions. The protein sequences were reverse-translated and optimized using DNAworks2.0 (ref. ^68^) with the *Saccharomyces cerevisiae* codon frequency table. Oligonucleotide pools encoding the designs were purchased from Agilent Technologies.

All libraries were amplified using Kapa HiFi polymerase (Kapa Biosystems) with a qPCR machine (Bio-Rad, CFX96). In detail, the libraries were first amplified in a 25-μl reaction and the PCR reaction was terminated when the reaction reached half-maximum yield to avoid overamplification. The PCR product was loaded onto a DNA agarose gel. The band with the expected size was cut out and DNA fragments were extracted using QIAquick kits (Qiagen). Then, the DNA product was reamplified as before to generate enough DNA for yeast transformation. The final PCR product was cleaned up with a QIAquick cleanup kit (Qiagen). For the yeast transformation step, 2–3 µg of linearized modified pETcon vector (pETcon3) and 6 µg of insert were transformed into the EBY100 yeast strain using a previously described protocol^69^.

DNA libraries for deep sequencing were prepared using the same PCR protocol, except the first step started from yeast plasmid prepared from 5 × 10^7^ to 1 × 10^8^ cells by Zymoprep (Zymo Research). Illumina adaptors and 6-bp pool-specific barcodes were added in the second qPCR step. Gel extraction was used to obtain the final DNA product for sequencing. All the different sorting pools were sequenced using Illumina NextSeq sequencing.

### Yeast surface display

*S.* *cerevisiae* EBY100 strain cultures were grown in C −Trp −Ura medium supplemented with 2% (w/v) glucose. For induction of expression, yeast cells were centrifuged at 6,000*g* for 1 min and resuspended in SGCAA medium supplemented with 0.2% (w/v) glucose at the cell density of 1 × 10^7^ cells per ml and induced at 30 °C for 16–24 h. Cells were washed with PBSF (PBS with 1% (w/v) BSA) and labeled with biotinylated targets using two labeling methods: with avidity and without avidity. For the with-avidity method, the cells were incubated with biotinylated target, together with anti-c-Myc fluorescein isothiocyanate (FITC; Miltenyi Biotech) and streptavidin–phycoerythrin (SAPE; Thermo Fisher). The concentration of SAPE in the with-avidity method was used at one quarter of the concentration of the biotinylated targets. For the without-avidity method, the cells were first incubated with biotinylated targets, washed and secondarily labeled with SAPE and FITC.

Cell sorting of labeled yeast pools was performed using a Sony SH800S cell sorter. Libraries of designs were sorted using the with-avidity method for the first few rounds of screening to exclude weak binder candidates, followed by several without-avidity sorts with different concentrations of targets. For SSM libraries, two rounds of with-avidity sorts were applied and, in the third round of screening, the libraries were titrated with a series of decreasing concentrations of targets to enrich mutants with beneficial mutations.

For yeast display characterization of individual designs, including competition assays, DNA sequences encoding the proteins of interest were purchased as Integrated DNA Technologies (IDT) E-Blocks, transformed into yeast cells and incubated in 96-well culture plates. Labeling with biotinylated dsDNA targets and SAPE/FITC was performed in a 96-well plate format. Of the 44 designs that were confirmed to bind their intended target in clonal yeast display experiments, (Extended Data Fig. 1), we categorized 14 with detectable binding to fewer than three of the 13 tested DNA targets (Extended Data Fig. 2) as specific binders and the remainder as nonspecific.

For yeast display competition assays, labeling was performed without avidity using 1 µM biotinylated dsDNA duplex oligos and an excess of 8 µM nonbiotinylated competitor dsDNA duplex oligos. As indicated in figure captions, some competition assays for higher-affinity binders were carried out with lower dsDNA oligo concentrations. Flow cytometry analysis was performed with an Attune NxT flow cytometer with autosampler. Flow cytometry data analysis was performed using custom Python code and the CytoFlow python package. For each individual sample, gating of the expression population was performed using the CytoFlow Gaussian mixture model and the ratio of SAPE channel intensity to FITC channel intensity (binding signal/expression signal) was calculated for all gated expression events of the sample.

### Deep sequencing analysis

The Pear program was used to assemble the fastq files from the deep sequencing runs. Translated, assembled reads were matched against the ordered design to determine the number of counts for each design in each pool. In each sequenced pool, binder enrichment was calculated by determining the percent of reads for each binder design in the pool and dividing this number by the same value in the naive expression sort pool. Designs were considered binders if >100-fold enrichment was observed in the last 1 µM with-avidity sort to the designed dsDNA target. For SSM libraries, apparent SC_50_ was estimated using the fitting procedure described in ref. ^6^.

### Protein expression and purification

DNA sequences encoding the proteins of interest were purchased as IDT E-Blocks and incorporated into plasmids using Golden Gate assembly. The plasmids were then transformed into BL21(DE3) competent *E.* *coli*. The transformation reactions were used to inoculate starter cultures in 5 ml or 25 ml of Terrific Broth (TB), supplemented with 1% (w/v) glucose and 50 mg L^−1^ kanamycin. After shaking overnight at 37 °C, the starter cultures were diluted 50-fold into 50 ml or 500 ml of TB with kanamycin. These cultures were incubated at 37 °C, shaking, until the optical density reached 0.6–0.8, at which point protein expression was induced by the addition of IPTG. The cultures were then further incubated overnight at 18 °C. Cells were harvested by centrifugation for 15 min at 3,000*g*, pellets were resuspended in lysis buffer (150 mM NaCl, 20 mM Tris-HCl, 0.5 mg ml^−1^ DNAse I and 1 mM PMSF, pH 8.0), the cells were lysed by sonication and the lysate was clarified by further centrifugation for 30 min at 20,000*g*. The supernatant was passed through Ni-NTA resin in a gravity column and then the resin was washed with 20 column volumes of high-salt wash buffer (2 M NaCl, 20 mM Tris-HCl and 20 mM imidazole, pH 8.0). Either the His-tagged protein was eluted with two column volumes of elution buffer (1 M NaCl, 20 mM Tris and 250 mM imidazole, pH 8.0) or the resin was further washed with five column volumes of SNAC buffer (100 mM CHES, 100 mM acetone oxime, 100 mM NaCl and 500 mM GnCl, pH 8.6), incubated in five column volumes of SNAC buffer + 0.2 mM NiCl_2_ on an orbital shaker at room temperature overnight and collected as the column flowthrough. Whether cleaved or not, the protein was concentrated to about 1 ml and loaded in 500-μl samples onto a Cytiva Superdex 75 Increase 10/300 GL gel filtration column equilibrated in buffer (1 M NaCl and 20 mM Tris-HCl, pH 8.0). Fractions containing monomeric protein were pooled and concentrated to about 200 μl. Protein concentrations were estimated spectroscopically by absorbance at 280 nm. For proteins with no tryptophan, tyrosine or cysteine residues, concentrations were approximated by Bradford reagent absorbance at 470 nm in comparison to BSA standards of known concentration.

### BLI

BLI binding data were collected on an Octet R8 (Sartorius) and processed using the instrument’s integrated software. Biotinylated dsDNA oligos were loaded onto streptavidin-coated biosensors (ForteBio) at 200 nM in PBS + 1% BSA + 0.05% Tween-20 for 6 min. Analyte proteins were diluted from concentrated stocks into the binding buffer. After baseline measurement in the binding buffer alone, the binding kinetics were monitored by dipping the biosensors in wells containing the target protein at the indicated concentration (association step) and then dipping the sensors back into baseline or buffer (dissociation). Data were analyzed and processed using ForteBio Data Analysis software v.9.0.0.14.

### Crystallization and structure determination

Purified DBP 48 was complexed with duplex DNAs, of varying duplex length and a single 5′ overhang base, to a final concentration of 176 µM DBP 48 and 233 µM duplex DNA. Complexes were screened for crystals in several broad matrix screens using a mosquito robot (SPT LabTech); then, possible hits were optimized in 24-well hanging drop trays with a 2-μl drop containing a 1:1 ratio of complex to well solution and equilibrated over 1 ml of well solution. A single diffraction-quality crystal was obtained with duplex DNA of length 10 bp with a single base overhang at either end of the duplex (5′-ACCTGACGCGA-3′, 3′-GGACTGCGCTT-5′) and a well condition containing 200 mM ammonium acetate, 100 mM sodium acetate at pH 4.6 and 28% PEG4000. The crystal was washed in well solution and then flash-cooled directly by plunging into liquid nitrogen. Data were collected at the Advanced Light Source in Berkeley on beam line 5.0.1 at a wavelength of 0.9762 Å and processed with DIALS^70^. Phases were determined through molecular replacement by searches with the original computational protein design and duplex DNA using Phaser^71^ in the PHENIX suite^72^. The top-scoring molecular replacement solutions were run through a round of refinement with PHENIX refine and further rounds of refinement with PHENIX refine and rebuilding with Coot^73^ were performed on the top-scoring structure. Data collection and refinement statistics are reported in Table 1.

**Table 1:** Data collection and refinement statistics

	DBP 48 + dsDNA^a^ (PDB 8TAC)
**Data collection**	
Space group	*P* 1 2_1_ 1
Cell dimensions	
*a*, *b*, *c* (Å)	28.352, 103.996, 32.825
*α*, *β*, *γ* (°)	90, 99.469, 90
Resolution (Å)	52–2.34 (2.424–2.34)
*R*_sym_ or *R*_merge_	0.1832 (0.2997)
*I*/σ*I*	5.38 (1.05)
Completeness (%)	99.57 (99.87)
Redundancy	6.2 (6.2)
**Refinement**	
Resolution (Å)	52–2.34 (2.424–2.34)
Number of reflections	7879 (771)
*R*_work_/*R*_free_	0.2441 (0.3196)/0.2793 (0.3653)
Number of atoms	
Protein	950
Ligands or ions	418
Water	68
*B* factors	
Protein	34.75
Ligand/ion	33.79
Water	27.77
R.m.s.d.	
Bond lengths (Å)	0.003
Bond angles (°)	0.58

^a^Values in parentheses are for the highest-resolution shell.

Statistics for the highest-resolution shell are shown in parentheses.

### uPBMs

uPBM experiments were carried out following the standard PBM protocol^38,39^. Briefly, we first performed primer extension to obtain dsDNA oligonucleotides on the microarray. Next, each microarray chamber was incubated with a 2% milk blocking solution for 1 h, followed by incubations with a PBS-based protein-binding mixture for 1 h and Alexa488-conjugated anti-His antibody (1:20 dilution; Qiagen, 35310) for 1 h. The array was gently washed as previously described^38^ and then scanned using a GenePix 4400A scanner (Molecular Devices) at 5-μm resolution. Data were normalized and processed with standard analysis scripts^38,39^.

### RFdiffusion-based design of DBP–TetR fusion linkers, homodimers and heterodimers

For TetR fusions, diffusion inputs were generated by manually aligning DBP domains (DBPs 48, 57 and 69) symmetrically relative to the TetR homodimer scaffold. A total of 10,000 RFdiffusion trajectories were run per input to generate rigid linkers between the DBP domains and the TetR homodimer scaffold. ProteinMPNN sequence design was performed on dimer diffusion outputs with tied positions between the two units and most residues of the DBP fixed, only allowing the design of DBP residues nearby the newly diffused linker region. Homodimer complexes were predicted with ESMFold because of the inability of AF2 to predict the MPNN-designed TetR backbones. Predicted structures were filtered on the r.m.s.d. of the predicted DBP regions to the input DBP domains and ESMFold pLDDT to select 96 designs across the three inputs.

For homodimer and heterodimer design, diffusion inputs were generated by aligning DBP domains (DBPs 9, 35opt, 57 and 69) symmetrically or asymmetrically onto DNA. A total of 10,000 RFdiffusion trajectories were run per input to generate *C*_2_-symmetric homodimers or asymmetric heterodimers between the DBP domains. ProteinMPNN sequence design was performed on diffusion outputs with tied positions between the two units (for homodimers) and most residues of the DBP fixed. Complexes were predicted with AF2 and filtered on r.m.s.d. of the predicted DBP regions to the input DBP domains and pLDDT to select 96 homodimer designs and 96 heterodimer designs.

### Transcriptional repression assays in *E.coli*

The pRF-TetR vector^40^ was used for transcriptional repression assays in *E.* *coli*. A new version of this vector (pRF-BsmB1) was constructed by first removing the LuxR gene and then replacing the TetR gene, its terminator sequence and regulated promoter with two BsmB1 cut sites such that new repressor variants and their associated promoters could be easily inserted by Golden Gate assembly^74^. For DBPs tethered with a flexible linker, a flexible linker was used to connect the C and N termini of two copies of the DBP (linker 1, KESGSVSSEQLAQFRSLD; linker 2, EGKSSGSGSESKST; linker 3, GGGGGGGG; linker 4, GSGSGSGSGSGSGSGS). Synthetic promoters were designed by inserting DNA-binding sites around the consensus −10 and −35 elements of the *E.* *coli* RNA polymerase promoter. Genes encoding the single-domain DBP, flexibly linked TetR fusions, homodimers and heterodimers were ordered as Twist synthetic gene fragments encoding the repressor gene (using Twist codon optimization), a transcriptional terminator and an associated synthetic promoter. Heterodimer constructs were encoded into bicistronic operons. Gene fragments were ordered containing BsmB1 cut sites on either end to allow for assembly into the modified pRF-BsmB1 vector. Upon Golden Gate assembly with the BsmB1 Type II-S restriction enzyme, plasmids were transformed into NEB 5α competent *E.* *coli* cells and streaked onto Luria–Burtani (LB) plates containing carbenicillin. All-by-all repressor constructs (Fig. 5c) were cloned by digestion with BsiWI-HF (New England Biolabs) and BbsI (New England Biolabs), followed by gel extraction of the backbone and promoter bands, ligation with T4 DNA ligase and transformation into NEB 5α competent *E.* *coli*.

Individual transformants were picked and verified by Sanger sequencing. Sequence verified colonies were inoculated into 200 µl of LB medium containing carbenicillin for overnight growth in 96-well round-bottom plates at 37 °C in a plate shaker. The following day, 2 µl of overnight cultures were transferred into a new plate with 200 µl of LB medium containing carbenicillin and appropriate concentrations of IPTG (1 mM in Fig. 5c) and grown for ~18 h in 96-well round-bottom plates at 37 °C. Flow cytometry analysis of cultures was performed with an Attune NxT flow cytometer with autosampler. Flow cytometry data analysis was performed using custom Python code and the CytoFlow python package. For each individual sample, gating was performed using the single component CytoFlow Gaussian mixture model and median BL1-A channel fluorescence was determined for all gated expression events of each sample. The median BL1-A channel fluorescence value of empty cells without a pRF vector was subtracted from the median BL1-A value of each sample. For each repressor variant in Fig. 5c and Extended Data Fig. 9d, fold repression was calculated from at least seven biological replicates as the ratio of median BL1-A channel fluorescence of the uninduced sample (background-subtracted) to the median BL1-A channel fluorescence of the induced sample (background-subtracted).

### Statistics and reproducibility

Statistical methods and the reproducibility of experiments are indicated in the respective figures. No data were excluded from the analyses. Data distribution was assumed to be normal but this was not formally tested. No statistical method was used to predetermine sample size but sample sizes were chosen to be consistent with those reported in previous publications^6^. The experiments were not randomized. The investigators were not blinded to allocation during experiments and analysis.

### Transcriptional activation in HEK293T cells

HEK293T cells purchased from the American Type Culture Collection expressing the PEmax were cultured in high-glucose DMEM (Gibco), supplemented with 10% FBS (Rocky Mountain Biologicals) and 1% penicillin–streptomycin (Gibco). Cells were grown with 5% CO_2_ at 37 °C. A total of 1 × 10^5^ cells were seeded on a 48-well plate 1 day before transfection. Enhancer plasmid and binder plasmid were mixed with a ratio of 2:1. Enhancer variants and background control were mixed with a ratio of 2:2:2:1. A total of 300 ng of plasmid was transfected using Lipofectamine 3000 (Thermo Fisher, L3000015), following the manufacturer’s protocol. Three synTF-specific recorders and 1 TCF^−^LEF^−^ recorder (negative control) were mixed with ratio 2:2:2:1 and cotransfected with synTFs into the HEK293T cells expressing PEmax. Three different spacings were tested—1 bp, 3 bp and 5 bp—between the palindromic binding motifs to maximize the recorder activity. Cells were harvested and analyzed 2 days after transfection. Genomic DNA was extracted on the basis of a protocol described previously^43^. Briefly, cells were lysed using freshly prepared lysis buffer (10 mM Tris-HCl pH 7.5, 0.05% SDS and 25 μg ml^−1^ protease (Thermo Fisher)) for each well. The genomic DNA mixture was incubated at 50 °C for 1 h, followed by an 80 °C enzyme inactivation step for 30 min. The DNA TAPE was amplified from the genomic DNA directly for next-generation sequencing. Recorded information was extracted using custom analysis code. Each enhancer has a unique barcode representing its activity. Transcription activation was measured as the fold change in the barcode abundance relative to the negative control barcode. All measurements were performed in triplicates. Error bars represent the s.d. of the mean relative barcode abundance.

### Reporting summary

Further information on research design is available in the Nature Portfolio Reporting Summary linked to this article.

## Online content

Any methods, additional references, Nature Portfolio reporting summaries, source data, extended data, supplementary information, acknowledgements, peer review information; details of author contributions and competing interests; and statements of data and code availability are available at https://doi.org/10.1038/s41594-025-01669-4.

## Supplementary information

Supplementary Information Supplementary Note 1 and Figs. 1–10.

Reporting Summary

Supplementary Table 1 Supplementary Tables 1–4.

Supplementary Data 1 Source data for Supplementary Fig. 2.

Supplementary Data 2 Source data for Supplementary Fig. 3.

Supplementary Data 3 Source data for Supplementary Fig. 5.

Supplementary Data 4 Source data for Supplementary Fig. 6.

Supplementary Data 5 Source data for Supplementary Fig. 9.

Supplementary Data 6 PDB design models for designs in Extended Data Fig. 1.

## Source data

Source Data Fig. 2 Statistical source data.

Source Data Fig. 3 Statistical source data.

Source Data Fig. 4 Statistical source data.

Source Data Fig. 5 Statistical source data.

Source Data Extended Data Fig. 1 Statistical source data.

Source Data Extended Data Fig. 2 Statistical source data.

Source Data Extended Data Fig. 3 Statistical source data.

Source Data Extended Data Fig. 4 Statistical source data.

Source Data Extended Data Fig. 5 Statistical source data.

Source Data Extended Data Fig. 6 Statistical source data.

Source Data Extended Data Fig. 8 Statistical source data.

Source Data Extended Data Fig. 9 Statistical source data.

Source Data Extended Data Fig. 10 Statistical source data.

## Data Availability

All underlying data for figures in the main text and extended data are supplied. Underlying data for the Supplementary Information, along with PDB files for design hits, are provided in the Supplementary Data 1–6. All sequencing data for yeast display sorting experiments and mammalian transcriptional activation assays were deposited to the National Center for Biotechnology Information Sequence Read Archive under BioProject PRJNA1014465. Protein-binding microarray data were deposited to the Gene Expression Omnibus under accession number GSE237017. The cocrystal structure of DBP 48 was deposited to the PDB under accession code 8TAC. Publicly available data were used for seeding bioinformatic searches and target DNA structures from the PDB under accession codes 1PER, 1BC8, 1YO5, 1L3L and 2O4A. The following publicly available sequence databases were used for bioinformatic searches: UniClust30 (https://uniclust.mmseqs.com), Uniref100 (https://www.uniprot.org/uniref) and JGI metagenome protein sequence database (https://genome.jgi.doe.gov/portal/). Source data are provided with this paper.

## Code availability

Custom scripts for running RIFgen/RIFdock and LigandMPNN are available from GitHub (https://github.com/cjg263/dbp_design). The Rosetta macromolecular modeling suite (https://www.rosettacommons.org) is freely available to academic and noncommercial users. Commercial licenses for the suite are available through the University of Washington Technology Transfer Office.
