# Supplementary material for: Computational design of sequence-specific DNA-binding proteins
Source: Nat Struct Mol Biol. 2025 Sep 12;32(11):2252–61. doi: 10.1038/s41594-025-01669-4 (PMC12618268; doi:10.1038/s41594-025-01669-4)
Supplement: Supplementary file 1 [file 41594_2025_1669_MOESM1_ESM.pdf]

nature structural & molecular biology

Supplementary information https://doi.org/10.1038/s41594-025-01669-4

Computational design of sequence-specific DNA-binding proteins

In the format provided by the authors and unedited

Supplementary Note 1:

Experimental characterization of a first round of designs generated using three-helix bundle scaffolds similar to those used for general protein binder design(*6*) showed that few bound their DNA targets and none bound specifically. To understand the reason for this failure, we carried out detailed structural inspection of these designs compared to natural DBPs. We observed that most natural DBPs make backbone amide-mediated hydrogen bond interactions with DNA phosphate oxygens (hereon called mainchain-phosphate hydrogen bonds) that were rare or absent in our docked and designed complexes. Thus, these scaffolds were unable to overcome the first DNA specific design challenge described in the design strategy.

Figs. S1 to S19

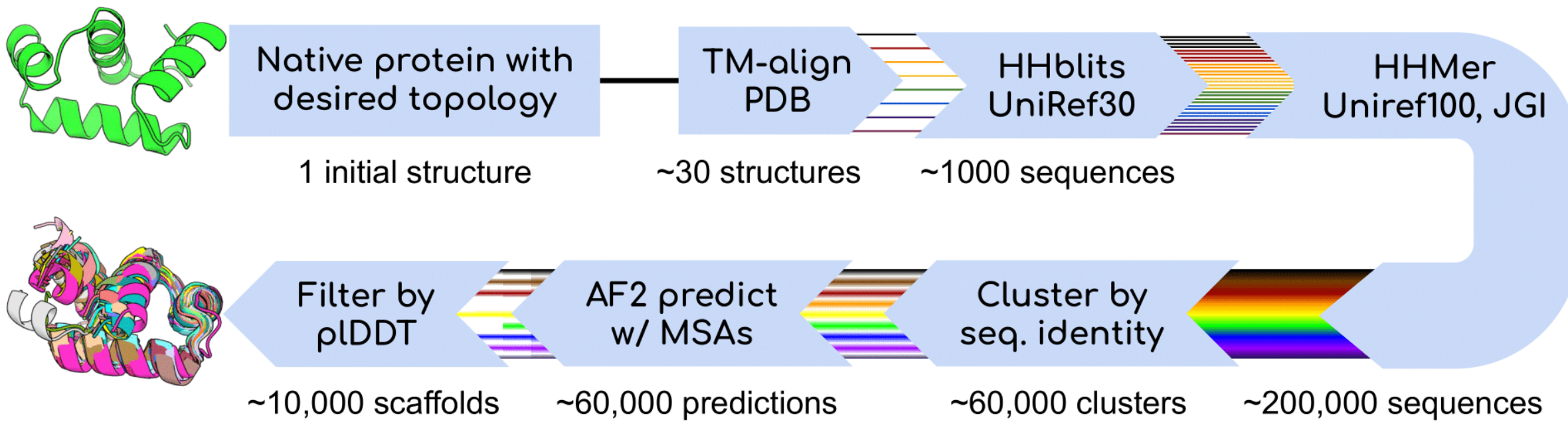

Supplementary Fig. 1. Overview of scaffold library generation. Scaffolds deposited in the Protein Data Bank (PDB) with structural similarity to selected template backbones were identified using TM-align(*29*). Amino acid sequences of identified protein scaffolds were used as seeds to generate multiple sequence alignments (MSAs) using an HHBlits(*53*) search of the UniRef30 database(*54*). Resulting MSAs were used for HMMer(*55*) searches of the JGI metagenome protein sequence databases(*56*) and the Uniref100 database(*54*). HMMer search results were clustered to < 70% sequence identity using MMSeqs2(*57*) and MSAs were generated from each clustered sequence using HHBlits. AlphaFold2(*28*) was used to predict structures for each sequence using the generated MSAs. Resulting scaffolds were filtered for high confidence AlphaFold2 pLDDT scores, TMscore to the input backbone templates, and Rosetta score. The above process was performed for two starting structures derived for PDB ID's 1L3L (*51*) 1PER (*52*) which were supplemented with additional AlphaFold2-predicted structures of transcription factor sequences identified from bacterial metagenomes using DeepTF(*58*). Approximate numbers correspond to the individual starting templates. PSSMs were generated for each scaffold using PSI-Blast(*59*) and custom code for use as constraints of Rosetta design. All final scaffolds are available for download.

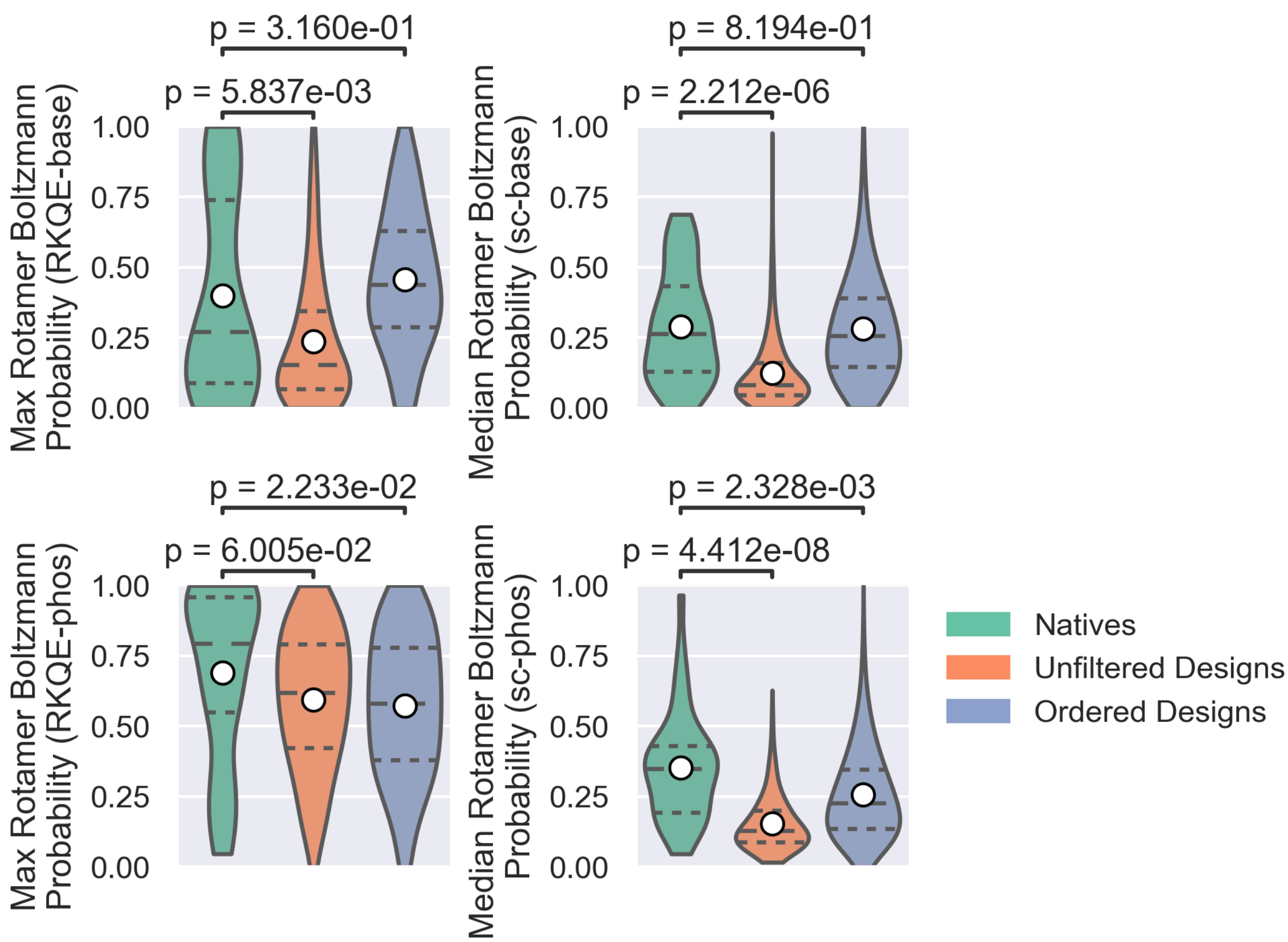

Supplementary Fig. 2. Native protein-DNA structures are enriched with highly preorganized residues forming hydrogen bonds with base atoms and the DNA phosphate backbone. The Rosetta RotamerBoltzmannWeight metric was calculated on natives (n=41 examples), unfiltered designs (n=2000 examples), and ordered designs (n=124,924 examples) as a proxy for sidechain preorganization. **Top: A,** Maximum RotamerBoltzmann probability among longer RKQE sidechains. **B,** Median RotamerBoltzmann probability for each design among all sidechains forming hydrogen bonds with bases. Among sidechains forming hydrogen bonds with base atoms, natives were significantly more preorganized than unfiltered designs. Ordered designs were filtered on the RotamerBoltzmann metric to achieve a distribution similar to natives. **Bottom: C,** Maximum RotamerBoltzmann probability among RKQE sidechains. **D,** Median RotamerBoltzmann probability among all sidechains forming hydrogen bonds with the phosphate backbone. Among sidechains forming hydrogen bonds with the phosphate backbone, both unfiltered and ordered designs were significantly less preorganized than natives. "Unfiltered Designs" were filtered by all other metrics except "Max RotamerBoltzmann Probability (RKQE-base)" and "Median RotamerBoltzmann Probability (sc-base)". "Ordered Designs" were filtered on "Max RotamerBoltzmann Probability (RKQE-base)" or "Median RotamerBoltzmann Probability (sc-base)" and comprise all designs that were tested by yeast display in this study. Violin plots show lower quartile, median, and upper quartile (dashed lines). White circles represent the mean of each distribution.

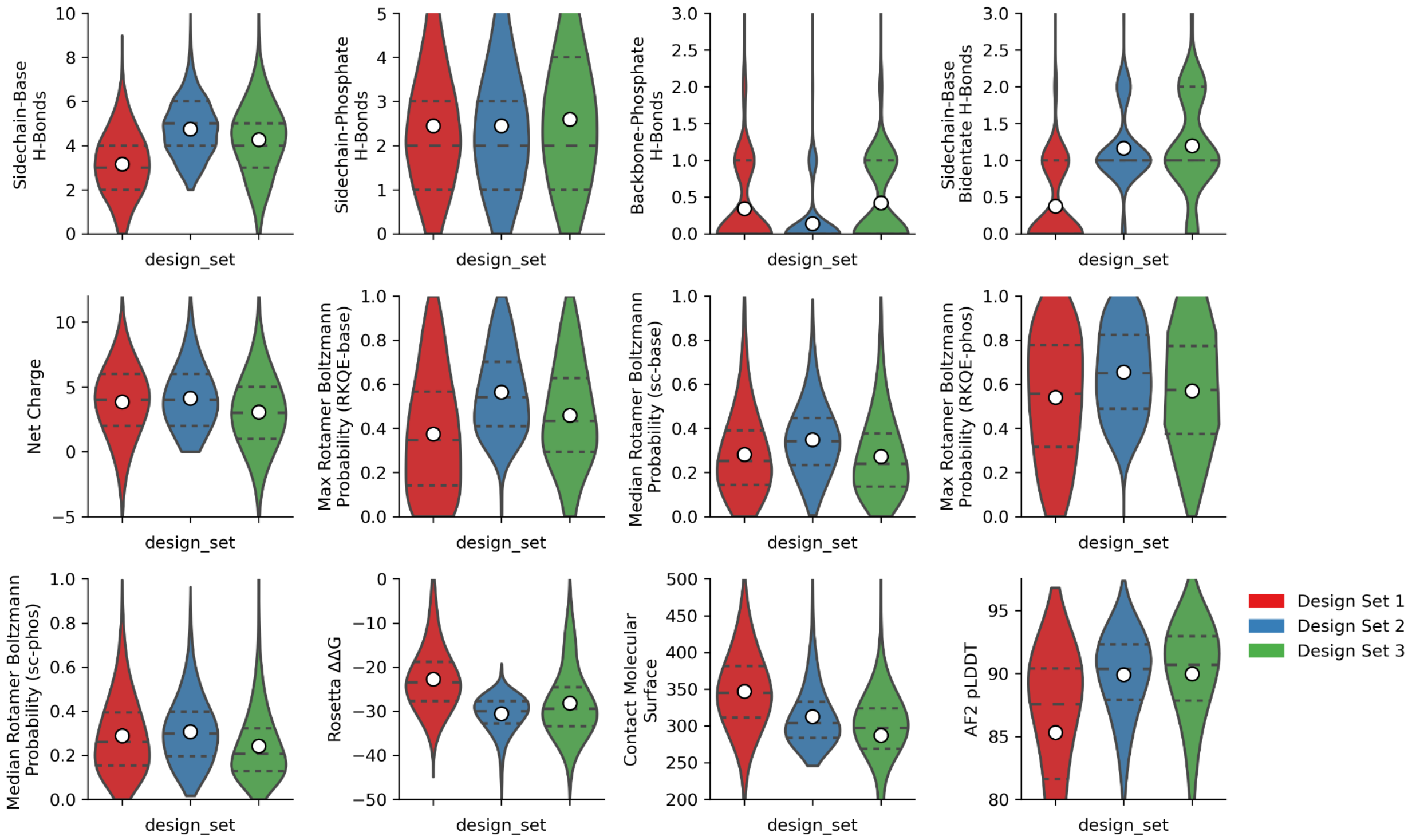

Supplementary Fig. 3. Distribution of metrics calculated on each ordered design set. Metrics were calculated on all designs after superposition of the AlphaFold2 predicted monomer onto the initial design complexes. Violin plots show lower quartile, median, and upper quartile (dashed lines). White circles represent the mean of each distribution. Design set 1 was produced using Rosetta sequence design and motif grafting, design set 2 was produced using LigandMPNN sequence design and motif grafting, and design set 3 was produced using LigandMPNN and Inpainting. White dots overlaid on violin plots represent the mean of each distribution while lower, middle, and upper lines represent first quartile, mean, and third quartile respectively.

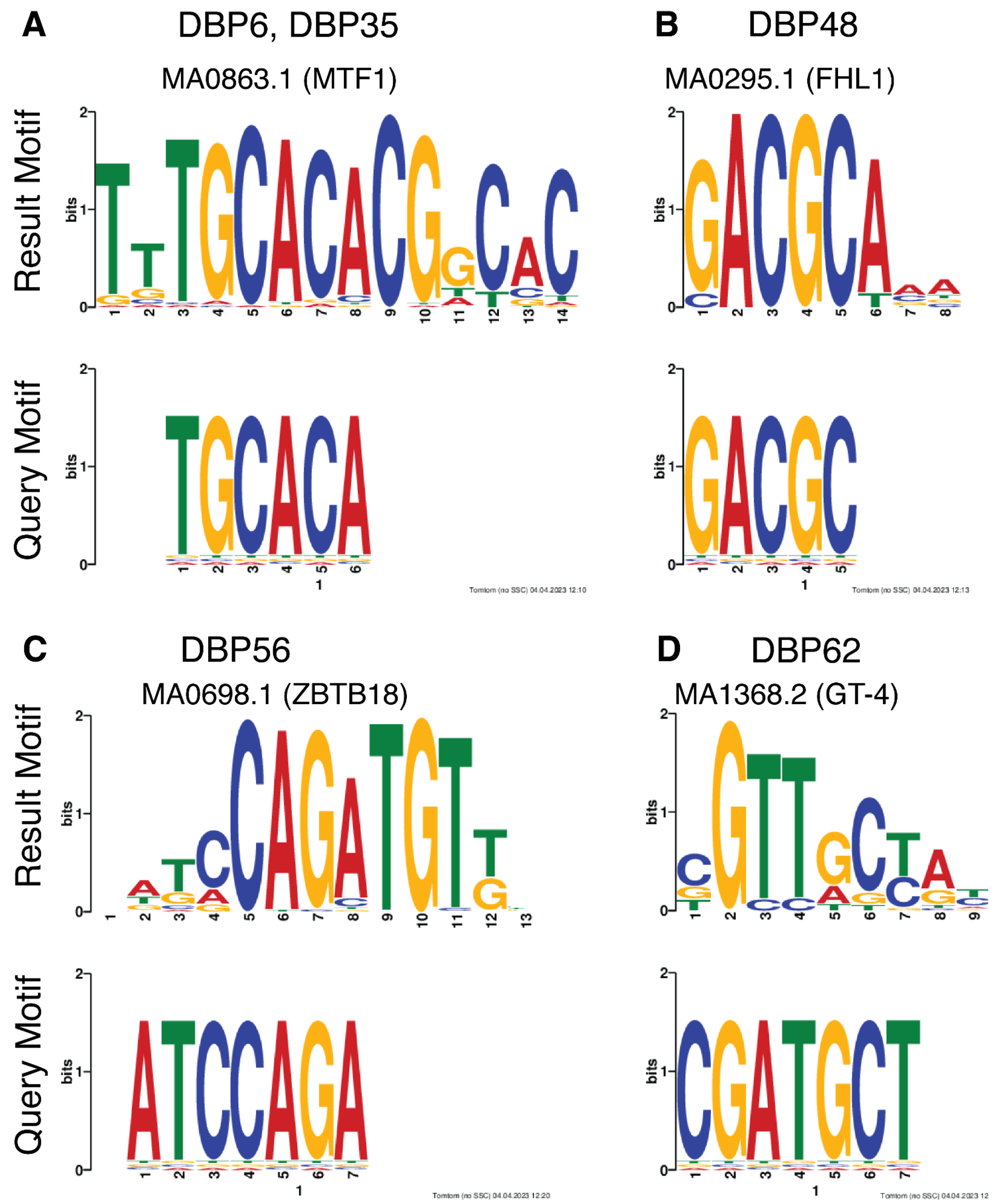

Supplementary Fig. 4. Comparison of designed DBP target motifs to JASPAR database. A-D, Comparison of the DNA binding site motifs found through motif searches of the JASPAR non-redundant transcription binding profile database. DBPs 6, 35, and 48 were found to bind highly similar sequences as the identified search hits in the JASPAR database; however, DBPs 56 and 62 were found to bind unique sequences compared to transcription factors with known specificity profiles. DBPs 6 and 35 were designed against the DNA sequence in the PDB structure 1L3L and thus were not expected to specifically bind a novel sequence. DBP48 was designed against a novel 14 bp sequence, but the experimentally verified 5 bp binding site motif was similar to the observed specificity of the FHL1 transcription factor.

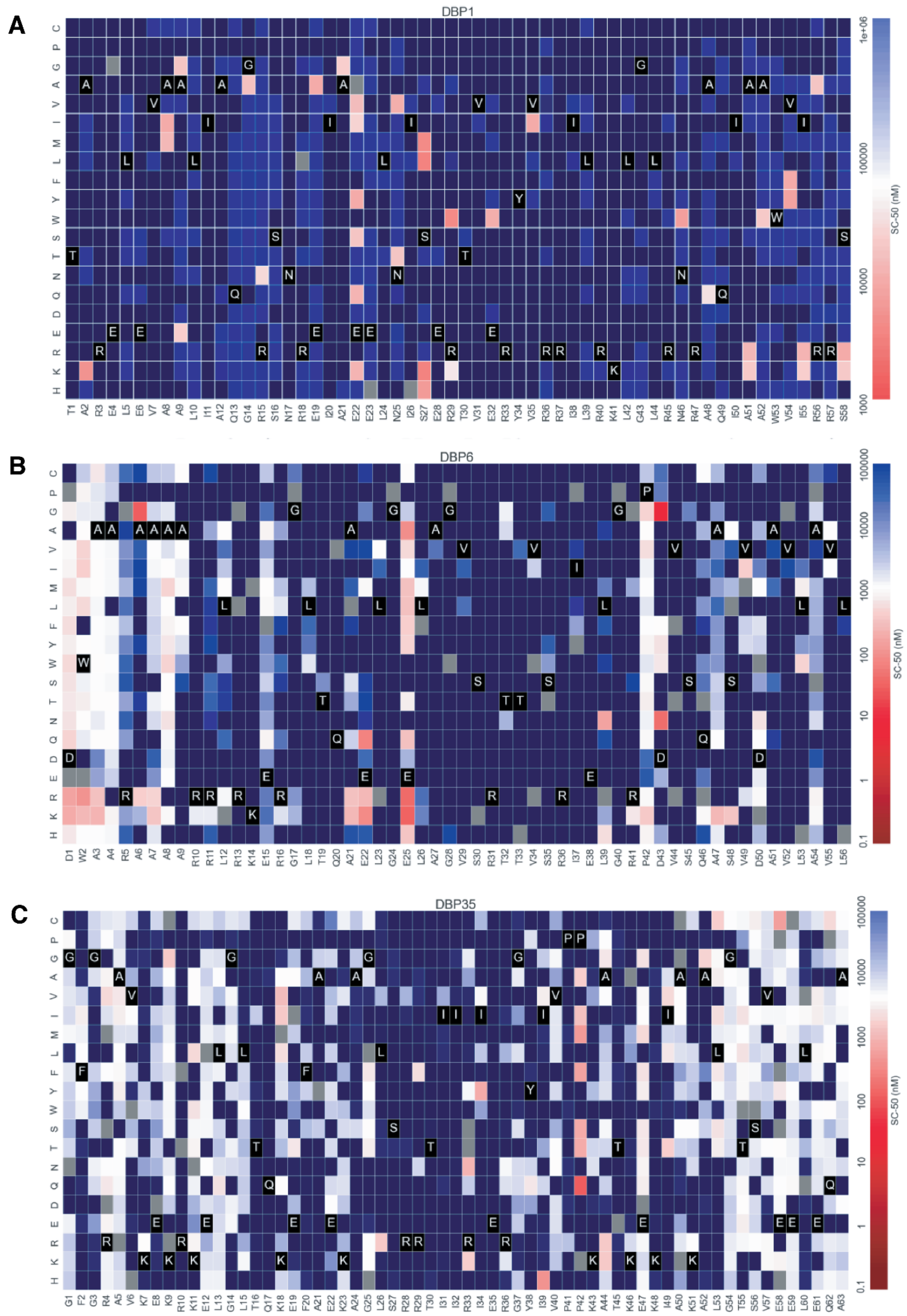

Supplementary Fig. 5. Full SSM maps for DBP1 (A), DBP6 (B), and DBP35 (C). Heat maps representing SC-50 values for single mutations in each design. The X-axis indicates the amino acid, while the Y-axis indicates the position and wild-type amino acid. Substitutions that are heavily depleted are shown in blue, and beneficial mutations are shown in red. The depletion of most substitutions in both the binding site and the core suggests that the design models are largely correct, whereas the enriched substitutions suggest routes to improving affinity.

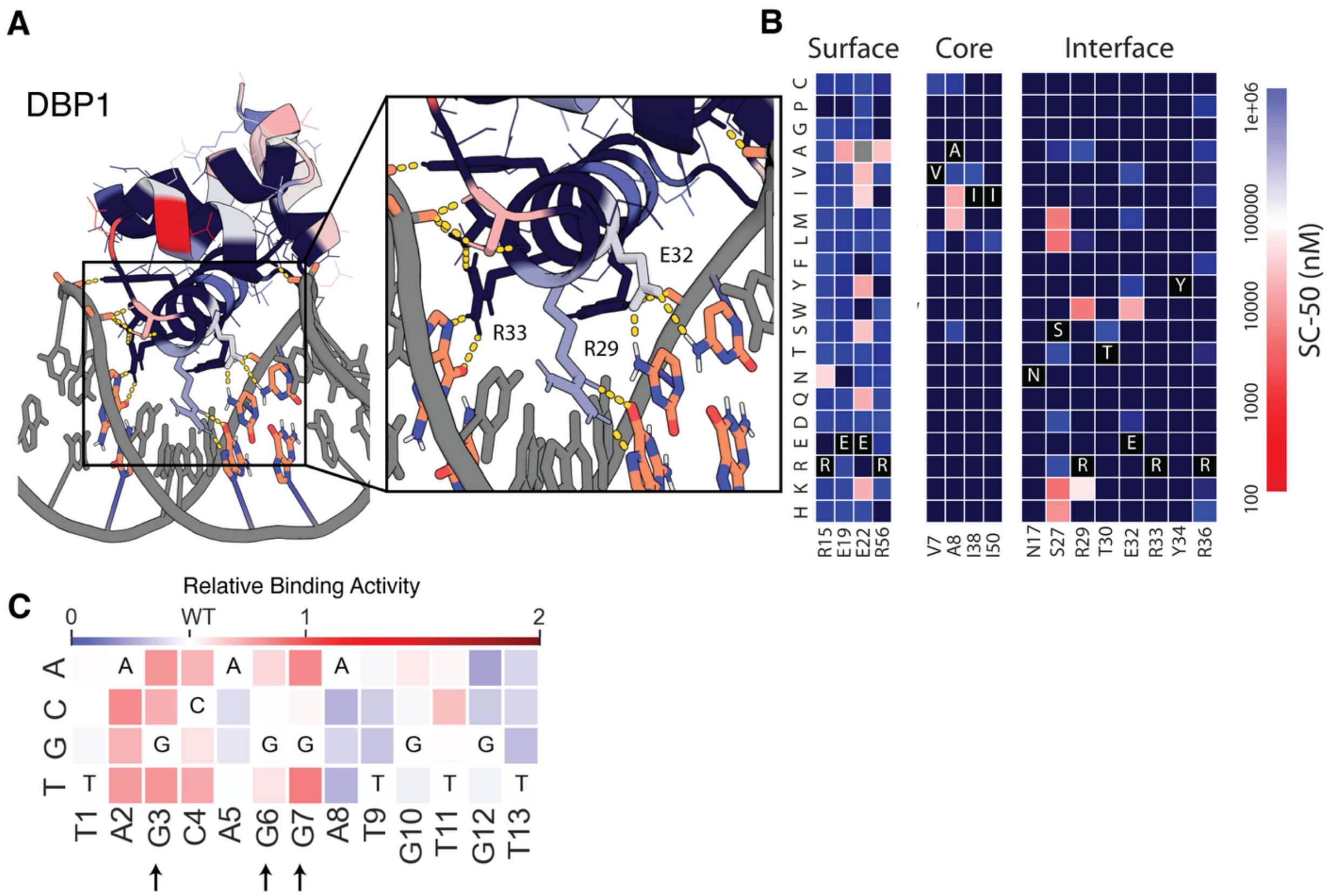

Supplementary Fig. 6. Close-up view of DBP1 SSM. A, Design model and close-up view of DBP1-DNA interface. **B,** Heat maps representing SC-50 values for single mutations in the design surface (left), core (middle) and the designed interface (right). Substitutions that are heavily depleted are shown in blue, and beneficial mutations are shown in red. The depletion of most substitutions in both the binding site and the core suggest that the design models are largely correct, whereas the enriched substitutions suggest routes to improving affinity. For DBP1, we noticed cases where interface mutations appeared to improve binding unexpectedly. R29W and E32W mutations in particular were found to improve binding. **C,** Clonal analysis by yeast display revealed that R29W substantially disrupted specificity when compared to the original design (**Extended Data Fig. 3**).

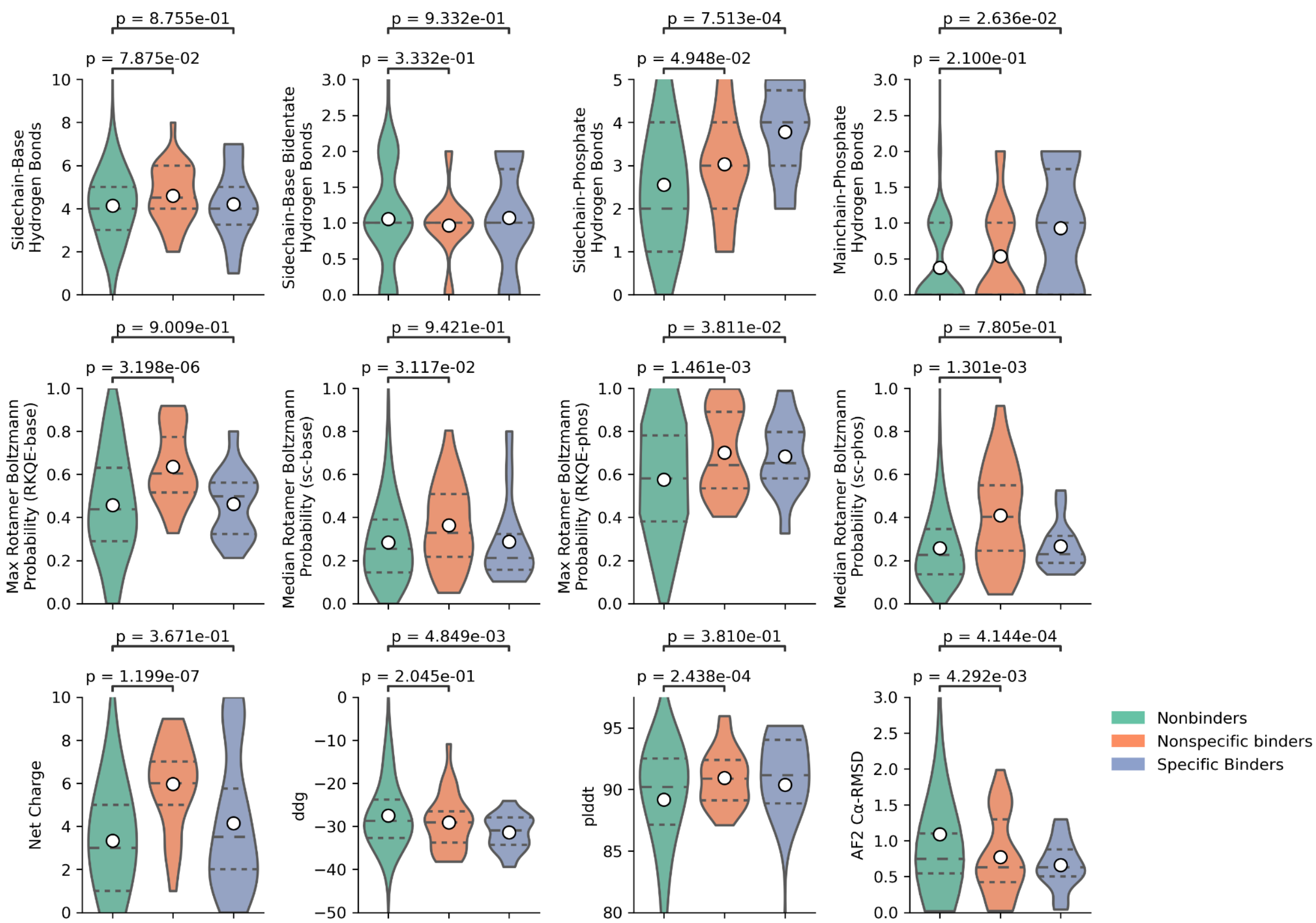

Supplementary Fig. 7. Power of computational metrics to predict binders. Metrics were calculated on all designs broken down into nonbinders (n=125,216 examples), nonspecific binders (n=30 examples), and specific binders (n=14 examples). Violin plots show lower quartile, median, and upper quartile (dashed lines). White circles represent the mean of each distribution. P-values were calculated using a two-sided Welch's t-test. Metrics associated with specific binders include number of sidechain- and backbone-phosphate hydrogen bonds, max RotamerBoltzmann probability of RKQE residues forming hydrogen bonds with the phosphate backbone, Rosetta ΔΔG, and Cα-RMSD of AlphaFold2 model to the initial design model. The different variations of the design pipeline were similarly successful in generating designs that bound their intended dsDNA oligo. While we found that generating large numbers of designs was most efficient and streamlined when using MPNN-based sequence design in conjunction with inpainting-based resampling, this approach had a lower overall success rate than the other design strategy variations, likely due to the comparatively more extensive search for suitable backbones when motif grafting with the entire scaffold library. The MPNN-based sequence design approach appeared to produce a slightly higher rate of successful designs than Rosetta-based sequence design and was also much more CPU efficient. Of the limited set of 7 designs assessed by microarray, the 4 MPNN-based designs were significantly more specific than the 2 Rosetta-based designs in the microarray context (**Extended Data Fig. 8**).

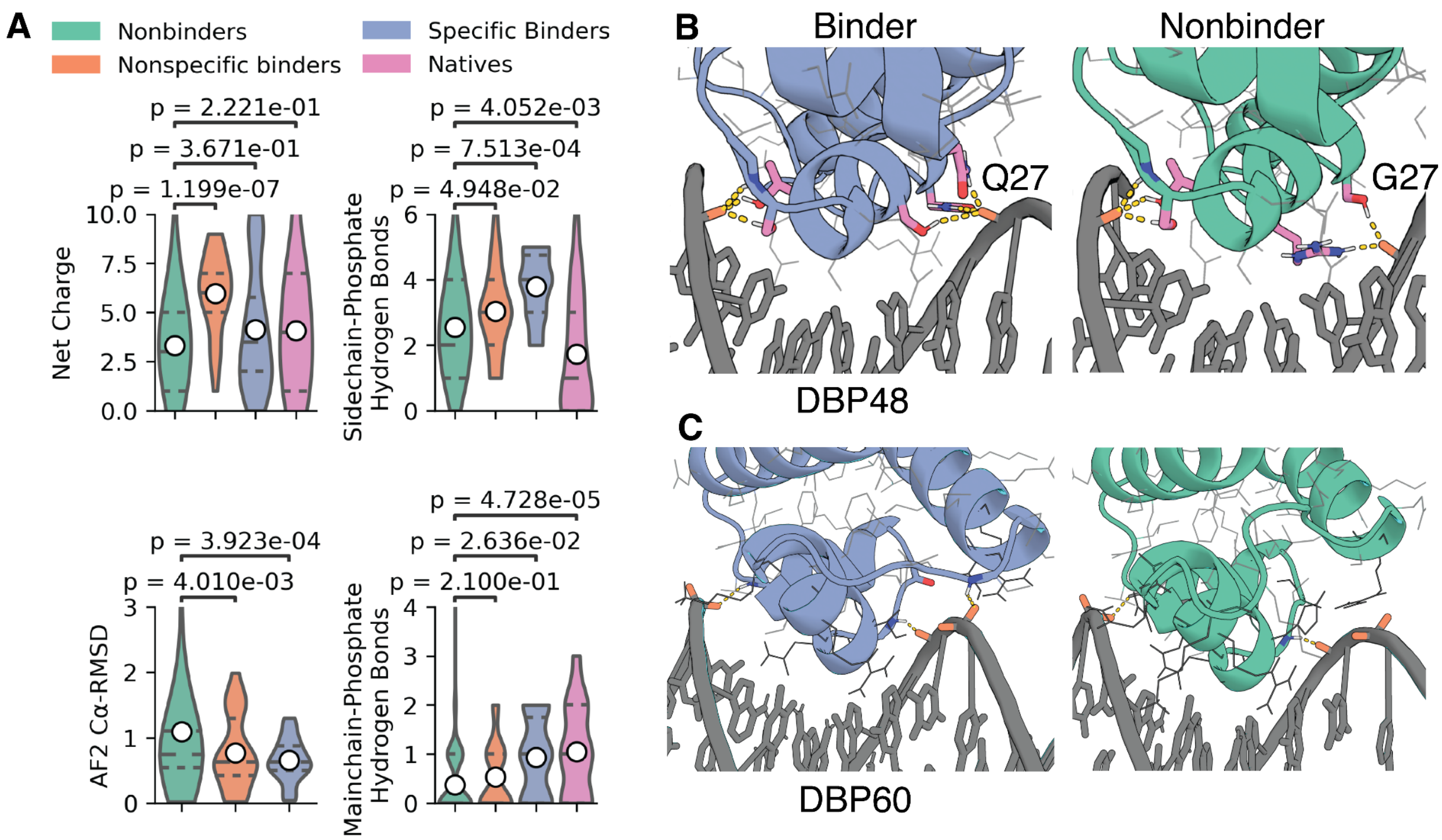

Supplementary Fig. 8. Mainchain-phosphate hydrogen bonds fix specificity and limit alternative target sites of DBP scaffolds. a, Comparison of the statistics calculated on nonbinders (n=125,216 examples), nonspecific binders (n=30 examples), and specific binders (n=14 examples), and native structures (n=41 examples). Violin plots show lower quartile, median, and upper quartile (dashed lines). White circles represent the mean of each distribution. P-values were calculated using a two-sided Welch's t-test. High net charge was correlated with nonspecific binder designs, while sidechain-phosphate and mainchain-phosphate hydrogen bonds were more correlated with specific binder designs. In both cases, designs having low Cα-RMSD of the initial design model to the AlphaFold model of the protein monomers was correlated with binding. Native structures have substantially more mainchain-phosphate hydrogen bonds than even the specific designs identified. **b,** Examples of a key sidechain-phosphate hydrogen bond in the highly-specific DBP48 design and a nearly identical nonbinding design containing a Q27G that disrupted the interaction. **c,** Example of a mainchain-phosphate hydrogen bond in highly-specific DBP60 and a nearly identical nonbinding design with the terminal helix moved away from the phosphate backbone.

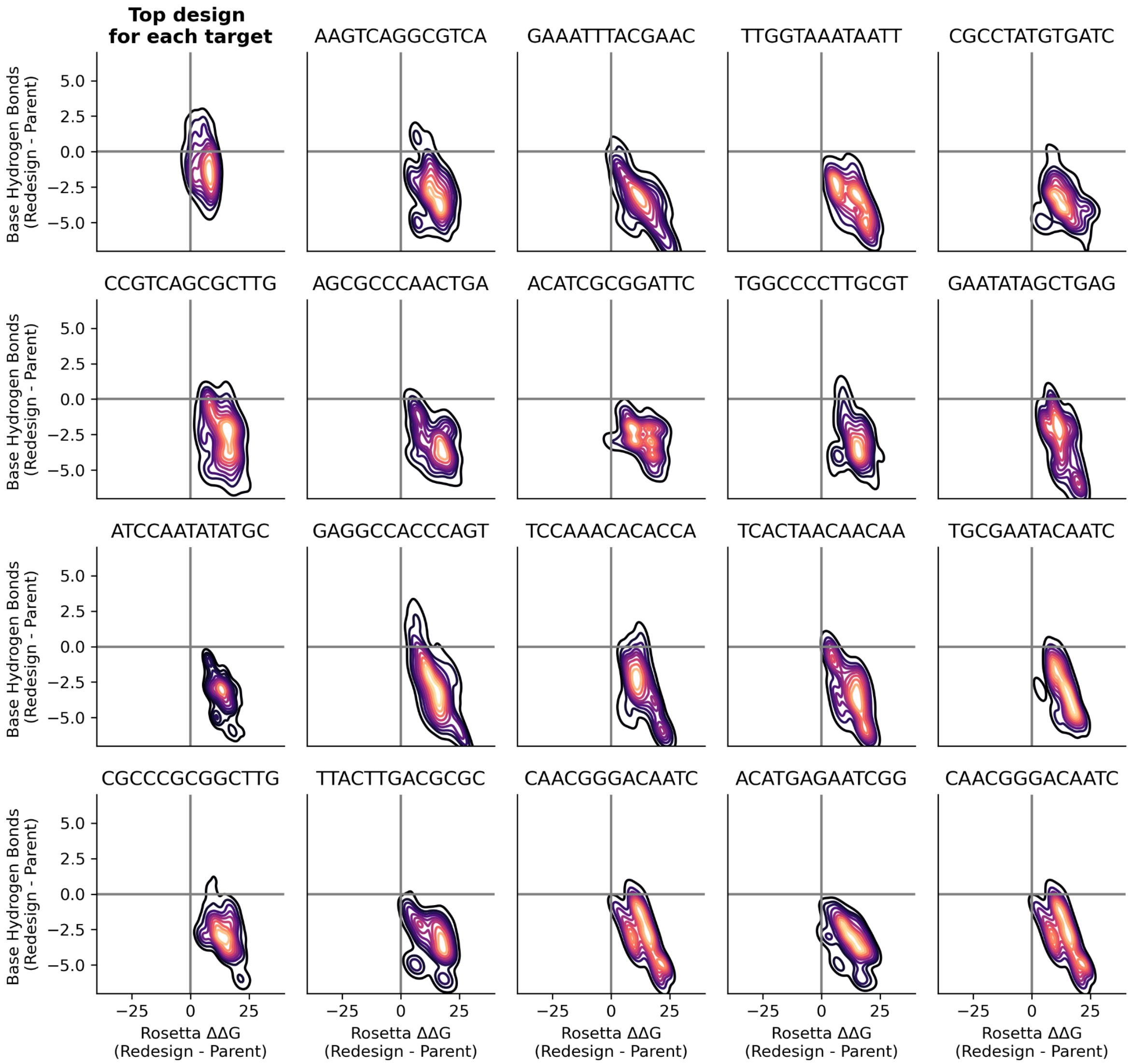

Supplementary Fig. 9. Starting dock restricts possible target sequences of DBP designs. MPNN-redesign of successful design scaffolds against alternative target sequences demonstrates that only few alternative sequences achieve statistics comparable to the parent scaffold complex. Fourteen design scaffold complexes were redesigned against 100 randomly generated DNA sequences after rethreading onto the design complex backbone, and 20 LigandMPNN sequences were generated for each design. Top left shows a kernel-density estimate (KDE) plot of re-designs with the lowest Rosetta ΔΔG for each of the 100 target sequences. The x-axis of each plot represents the difference of Rosetta ΔΔG of each redesign from its respective parent complex, while the y-axis represents the difference in the number of base-specific hydrogen bonds. Redesigns for 2 out of 100 target sequences achieved a Rosetta ΔΔG and number of hydrogen bonds to bases comparable to their respective starting scaffold complexes. The remaining subplots show KDE plots of redesigns generated for 19 additional randomly selected sequences.

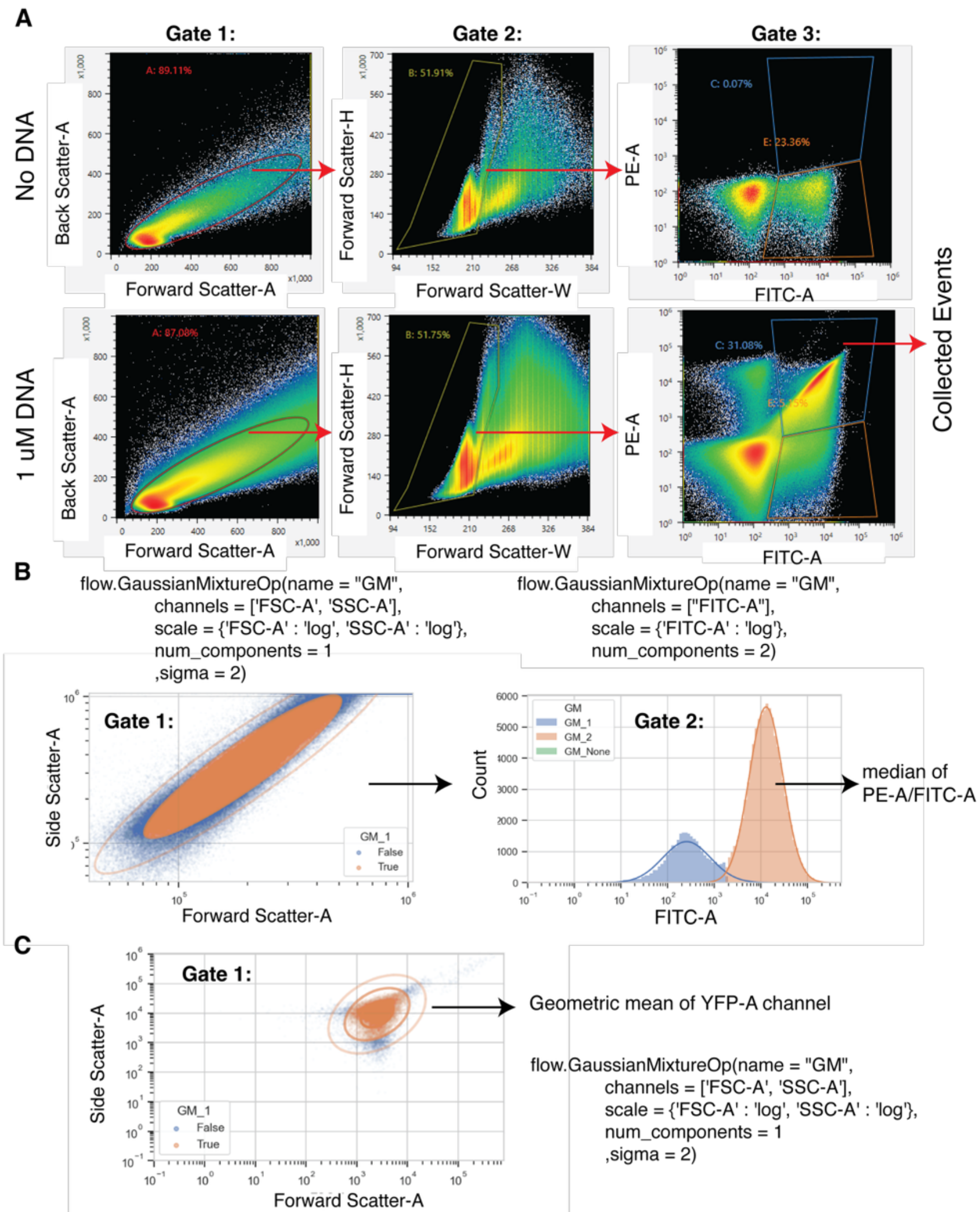

Supplementary Fig. 10. Example gating strategies for flow cytometry analysis. a, Gating for yeast display cell sorting. Gate 1 was set around the center of the distribution on BSC-A/FSC-A. Gate 2 was used to select single cells on the FSC-H/FSC-W distribution from the population selected in gate1. Gate 3 was used to select the FITC-A and PE-A double positive events from the population in gate 2 that were collected in sorting experiments. **b**, Example images of the automated gating strategy used for all clonal titration and competition yeast display assays performed on the Attune NxT. In gate 1, the GaussianMixtureOp function in the Cytoflow software was used to automatically select the center (orange). In gate 2, the GaussianMixtureOp function was used to select the FITC-A positive cells (orange) from the population selected in gate 1. The FITC-A positive cells from gate 2 were used to extract the median of PE-A/FITC-A values for analysis of each sample in Figure 2b, Figure 4b, Extended Data Figure 1, Extended Data Figure 2, Extended Data Figure 3, Extended Data Figure 9d-I, and Supplemental Figure 6c. **c**, Example images of the automated gating strategy used for E. coli transcriptional repression assays performed on the Attune NxT. The GaussianMixtureOp function in the Cytoflow software was used to automatically select the single cell population (orange) from which the geometric mean of the YFP channel was extracted for analysis in Figure 5 and Extended Data Figure 10b-g. Functions for performing the Gaussian Mixture Operation on both E. coli and yeast samples are shown above each plot.
