# Supplementary material for: Computational design of sequence-specific DNA-binding proteins
Source: Nat Struct Mol Biol. 2025 Sep 12;32(11):2252–61. doi: 10.1038/s41594-025-01669-4 (PMC12618268; doi:10.1038/s41594-025-01669-4)
Supplement: Supplementary file 2 [file 41594_2025_1669_MOESM2_ESM.pdf]

nature portfolio

Corresponding author(s): Cameron Glasscock, David Baker

Last updated by author(s): July 2, 2025

Reporting Summary

Nature Portfolio wishes to improve the reproducibility of the work that we publish. This form provides structure for consistency and transparency in reporting. For further information on Nature Portfolio policies, see our Editorial Policies and the Editorial Policy Checklist.

Statistics

For all statistical analyses, confirm that the following items are present in the figure legend, table legend, main text, or Methods section.

n/a	Confirmed	
☐	☒	The exact sample size (*n*) for each experimental group/condition, given as a discrete number and unit of measurement
☐	☒	A statement on whether measurements were taken from distinct samples or whether the same sample was measured repeatedly
☐	☒	The statistical test(s) used AND whether they are one- or two-sided<br>*Only common tests should be described solely by name; describe more complex techniques in the Methods section.*
☐	☒	A description of all covariates tested
☐	☒	A description of any assumptions or corrections, such as tests of normality and adjustment for multiple comparisons
☐	☒	A full description of the statistical parameters including central tendency (e.g. means) or other basic estimates (e.g. regression coefficient) AND variation (e.g. standard deviation) or associated estimates of uncertainty (e.g. confidence intervals)
☐	☒	For null hypothesis testing, the test statistic (e.g. *F*, *t*, *r*) with confidence intervals, effect sizes, degrees of freedom and *P* value noted<br>*Give P values as exact values whenever suitable.*
☒	☐	For Bayesian analysis, information on the choice of priors and Markov chain Monte Carlo settings
☒	☐	For hierarchical and complex designs, identification of the appropriate level for tests and full reporting of outcomes
☒	☐	Estimates of effect sizes (e.g. Cohen's *d*, Pearson's *r*), indicating how they were calculated

Our web collection on statistics for biologists contains articles on many of the points above.

Software and code

Policy information about availability of computer code

Data collection	Rosetta Macromolecular Modeling Suite 3.14; RifDock (https://github.com/rifdock/rifdock); LigandMPNN (https://github.com/dauparas/LigandMPNN), TMAlign 20180822 (https://zhanggroup.org/TM-align/), hh-suite3 (https://github.com/soedinglab/hh-suite), hmmer-3.4 (http://hmmer.org/download.html), MMseqs2 (https://github.com/soedinglab/MMseqs2), AlphaFold2 v2 (https://github.com/google-deepmind/alphafold), DeepTF (https://github.com/Min-Sheng/DeepTF), blast+ 2.11.0 (https://www.ncbi.nlm.nih.gov/books/NBK2590/), X3DNA v.4.8 (https://x3dna.org),
Data analysis	Python3.8; ForteBio Data Analysis Software Version 9.0.0.14; cytoflow v1.2.2 (https://cytoflow.readthedocs.io/en/stable/)

For manuscripts utilizing custom algorithms or software that are central to the research but not yet described in published literature, software must be made available to editors and reviewers. We strongly encourage code deposition in a community repository (e.g. GitHub). See the Nature Portfolio guidelines for submitting code & software for further information.

April 2023

Data

Policy information about availability of data

All manuscripts must include a data availability statement. This statement should provide the following information, where applicable:

- Accession codes, unique identifiers, or web links for publicly available datasets
- A description of any restrictions on data availability
- For clinical datasets or third party data, please ensure that the statement adheres to our policy

All underlying data for figures in the main text, extended data are supplied. Underlying data for the supplement, along with PDB files for design hits, are provided in the supplementary data files. All sequencing data for yeast display sorting experiments and mammalian transcriptional activation assays have been deposited to the NCBI SRA (accession #: PRJNA1014465). Protein-binding microarray data is deposited to GEO (accession #: GSE237017). The co-crystal structure of DBP48 has been deposited in RCSB as PDB ID 8TAC. Publicly available data from the PDB (https://www.rcsb.org) were used for seeding bioinformatic searches and target DNA structures (accession codes 1PER, 1BC8, 1YO5, 1L3L, 2O4A). The following publicly available sequence databases were used for bioinformatic searches: UniClust30 (https://uniclust.mmseqs.com), Uniref100 (https://www.uniprot.org/uniref), JGI metagenome protein sequence database (https://genome.jgi.doe.gov/portal/).

Research involving human participants, their data, or biological material

Policy information about studies with human participants or human data. See also policy information about sex, gender (identity/presentation), and sexual orientation and race, ethnicity and racism.

Reporting on sex and gender	N/A
Reporting on race, ethnicity, or other socially relevant groupings	N/A
Population characteristics	N/A
Recruitment	N/A
Ethics oversight	N/A

Note that full information on the approval of the study protocol must also be provided in the manuscript.

Field-specific reporting

Please select the one below that is the best fit for your research. If you are not sure, read the appropriate sections before making your selection.

☒ Life sciences ☐ Behavioural & social sciences ☐ Ecological, evolutionary & environmental sciences

For a reference copy of the document with all sections, see nature.com/documents/nr-reporting-summary-flat.pdf

Life sciences study design

All studies must disclose on these points even when the disclosure is negative.

Sample size	Sample sizes are noted in the figure legend of each experiment. For design libraries, 5,000 to 10,000 designs were ordered for each targeting sequence and this depends on the Agilent Oligo library size. No statistical method was used to determine the total number of designs to be experimentally tested. The numbers are chosen because the size of an Agilent Oligo Pool is 15,000 or 60,000.
Data exclusions	There is no data exclusion in this study.
Replication	Replicate data were collected as noted in the figure legend for each experiment and all attempts at replication were succesful. When results are reported as the mean of replicate data points, individual replicate data are shown in the supplemental info. For yeast display and transcriptional regulation experiments replicates are unique biological replicates, for microarray data replicates are technical replicates from the same protein purification batch.
Randomization	Randomization is not relevant to this study, as the performed experiments are minimally affected by environmental or operator variability.
Blinding	Blinding is not relevant to this study, as the values we measured in experiments are quantitative and do not need subject judgement

Reporting for specific materials, systems and methods

We require information from authors about some types of materials, experimental systems and methods used in many studies. Here, indicate whether each material, system or method listed is relevant to your study. If you are not sure if a list item applies to your research, read the appropriate section before selecting a response.

Materials & experimental systems

n/a	Involved in the study
☐	☒ Antibodies
☐	☒ Eukaryotic cell lines
☒	☐ Palaeontology and archaeology
☒	☐ Animals and other organisms
☒	☐ Clinical data
☒	☐ Dual use research of concern
☒	☐ Plants

Methods

n/a	Involved in the study
☒	☐ ChIP-seq
☐	☒ Flow cytometry
☒	☐ MRI-based neuroimaging

Antibodies

Antibodies used	anti-c-myc (Immunology Consultants CMYC45F, 1:100 dilution)
Validation	Validation was performed by the vendor (https://www.icllab.com/media/catalog/product//pdf/PP_CMYC-45F_1.pdf) and confirmed to react with EQKLISEEDL as determined by ELISA and IEP techniques.

Eukaryotic cell lines

Policy information about cell lines and Sex and Gender in Research

Cell line source(s)	HEK293T (ATCC)
Authentication	All cell lines were used as received without further authentication
Mycoplasma contamination	Cell lines were not detected for mycoplasma contamination
Commonly misidentified lines (See ICLAC register)	Commonly misidentified cell lines were not used in this study

Plants

Seed stocks	N/A
Novel plant genotypes	N/A
Authentication	N/A

Flow Cytometry

Plots

Confirm that:

☒ The axis labels state the marker and fluorochrome used (e.g. CD4-FITC).

☒ The axis scales are clearly visible. Include numbers along axes only for bottom left plot of group (a 'group' is an analysis of identical markers).

☒ All plots are contour plots with outliers or pseudocolor plots.

☒ A numerical value for number of cells or percentage (with statistics) is provided.

Methodology

Sample preparation	Yeast cells are incubated with the target protein and then labeled with anti-Myc antibody conjugated with FITC and Streptavidin with PE. The cells were washed with PBSF. See methods for experimental deails. For E. coli experiments, cells were washed with PBSF and directly measured in the flow cytometer for YFP expression.
Instrument	Sony SH800, ThermoFisher Attune NXT

Software	CytoFlow
Cell population abundance	Yes
Gating strategy	Cells labeled without the target protein were used as negative control and all the cells showing binding signal were collected. Example code for data analysis and gating strategy are provided in Supplementary Information Figure 10. At least 15,000 events were collected for all analytical samples.

☒ Tick this box to confirm that a figure exemplifying the gating strategy is provided in the Supplementary Information.
